# Global Gene Expression Analysis of Long-Term Stationary Phase Effects in *E. coli* K12 MG1655

**DOI:** 10.1371/journal.pone.0096701

**Published:** 2014-05-23

**Authors:** Kotakonda Arunasri, Mohammed Adil, Pathan Akbar Ali Khan, Sisinthy Shivaji

**Affiliations:** CSIR-Centre for Cellular and Molecular Biology, Hyderabad, India; University of Hyderabad, India

## Abstract

Global gene expression was monitored in long-term stationary phase (LSP) cells of *E. coli* K12 MG1655 and compared with stationary phase (SP) cells that were sub-cultured without prolonged delay to get an insight into the survival strategies of LSP cells. The experiments were carried out using both LB medium and LB supplemented with 10% of glycerol. In both the media the LSP cells showed decreased growth rate compared to SP cells. DNA microarray analysis of LSP cells in both the media resulted in the up- and down-regulation of several genes in LSP cells compared to their respective SP cells in the corresponding media. In LSP cells grown in LB 204 genes whereas cells grown in LB plus glycerol 321 genes were differentially regulated compared to the SP cells. Comparison of these differentially regulated genes indicated that irrespective of the medium used for growth in LSP cells expression of 95 genes (22 genes up-regulated and 73 down-regulated) were differentially regulated. These 95 genes could be associated with LSP status of the cells and are likely to influence survival and growth characteristics of LSP cells. This is indeed so since the up- and down-regulated genes include genes that protect *E. coli* LSP cells from stationary phase stress and genes that would help to recover from stress when transferred into fresh medium. The growth phenotype in LSP cells could be attributed to up-regulation of genes coding for insertion sequences that confer beneficial effects during starvation, genes coding for putative transposases and simultaneous down-regulation of genes coding for ribosomal protein synthesis, transport-related genes, non-coding RNA genes and metabolic genes. As yet we still do not know the role of several unknown genes and genes coding for hypothetical proteins which are either up- or down-regulated in LSP cells compared to SP cells.

## Introduction

Bacterial cells when inoculated into a fresh medium exhibit a growth cycle with five phases [1], [2]. Initially the bacteria exhibit a lag phase at which cell number is not increased. The lag phase is followed by the log or the exponential phase when the cell number increases rapidly. These exponentially growing bacteria due to depletion of nutrients, accumulation of waste products and high cell density enter into the stationary phase during which no increase in cell number is observed. Nutrient-depleted stationary-phase cultures eventually enter a death phase when 90–99% of the population dies releasing nutrients that could be scavenged by the surviving bacteria. Such bacteria could survive for prolonged periods (months or even years) and this phase has been termed as long-term stationary phase [1]. This phase is characterized by alternating peaks of growth and death [3]. Thus study of cells in the long-term stationary phase (LSP) would help to unravel strategies for long-term survival of bacteria [1]. Cells in order to sustain life under these starving conditions form dormant spores, aggregates and/or fruiting bodies [4]. In addition, the cells may undergo a change in morphology from rod-shaped to coccoid cells as in *Escherichia coli* [5]. The cell membrane also becomes less permeable and less fluid [6].

One important molecular change that becomes obvious during the long-term stationary phase is the decline in the protein synthetic capacity of the cells, thus resulting in surplus ribosomes that are no longer involved in translation. These surplus ribosomes are either degraded as in *Salmonella* [7] or modified as in *E. coli* [8]. The decrease in protein synthesis in *E. coli* has been attributed to a decrease in concentration of the three initiation factors, particularly IF3 [9]. During prolonged starvation which occurs in LSP, cells exhibit a phenomenon termed as ‘growth advantage in stationary phase’ (GASP), where the surviving cells exhibit a growth advantage over the wild type cells [1]. Survival of GASP cells may be due to the expression of several survival genes in long-term stationary phase in order to combat stress due to depletion of nutrients and other environmental factors [5] or due to adaptive mutations in cells that are exposed to stationary phase for long duration [1].

The primary aim of the study is to monitor changes at gene expression level in a LSP culture of *E. coli* K12 MG1655 kept for 28 days at room temperature and subsequently grown to a mid logarithmic phase of growth in a fresh medium. Comparison of the global gene expression of these LSP cells with those of stationary phase cells that were sub-cultured without prolonged delay would provide insight into the survival strategies of LSP cells. The results indicated that expression of genes is dependent on the physiological state (stationary phase versus LSP) and the results are in accordance with earlier reports that gene expression is controlled by the physiological state of the cell [10], [11]. In addition, DAVID (Database for Annotation, Visualization and Integrated Discovery) analysis revealed various geneontology term enrichments pertaining to LSP. Finally, the comparative differential gene expression data of *E. coli* grown in LB broth versus *E. coli* grown in LB broth supplemented with glycerol broth provided insight into the effects pertaining to the LSP in *E. coli*.

## Materials and Methods

### Bacterial Strain and Growth Conditions

Stock cultures of *E. coli* K12 MG1655 (henceforth *E. coli*) were maintained on Luria-Bertani (LB) agar plates (containing 10 g peptone, 10 g NaCl, 5 g yeast extract and 2% (w/v) agar in 1000 ml distilled water) at 30°C. To develop the inoculums, a single colony of *E. coli* from the LB agar plate was inoculated into LB broth (LB agar minus agar) and incubated for growth under shaking at 150 r.p.m and at 30°C. *E. coli* growth was monitored by measuring absorbance at 600 nm using a spectrophotometer (Shimadzu UV-VIS 1700, North America). *E. coli* grown to a OD of 0.8 (10^6^ CFU/ml) was used as inoculum for all the experiments unless otherwise mentioned.

### Growth of *E. coli* in Two Different Media

**Figure 1:**
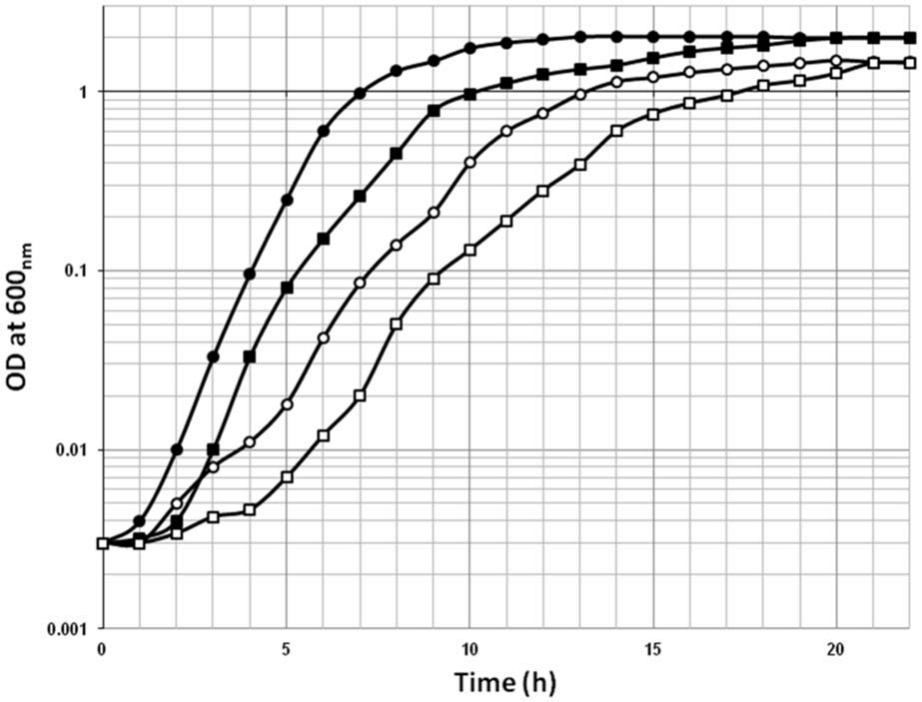
Growth of stationary phase culture of *E. coli* (O,•) and long-term stationary phase culture of *E. coli* which was held for 28 days at room temperature (□, ▪) that were sub-cultured for growth at 30°C in Luria-Bertani (LB) broth in the presence (O, □) and absence (•, ▪) of glycerol (10%).

*E. coli* was grown aerobically in two different media namely Luria-Bertani broth (LB) and LB broth supplemented with 10% (v/v) glycerol (hereafter LB + glycerol) at 30°C in an incubator shaker with continuous shaking (150 r.p.m). When the cells reached 0.8 OD _600nm_, the culture was centrifuged and the pellet was suspended into a fresh medium to a concentration of 10^6^ CFU/5 ml. These cells were then held for 28 days at room temperature without shaking. After 28 days the cells (OD at 600 nm was recorded as 1.8 and 1.5 for both LB broth and LB + glycerol grown *E. coli* cultures respectively) were inoculated (5% v/v containing10^6^ CFU/ml) into 100 ml of LB broth and 100 ml of LB + glycerol respectively and growth was monitored by measuring absorbance at 600 nm. For this purpose aliquots of 0.5 ml were withdrawn at regular intervals of time and OD at 600 nm was recorded and the growth curves plotted (Figure 1). Growth studies were conducted in three replicates for each culture condition. To compare the growth of LSP *E. coli* cells with stationary phase cells (SP), overnight grown *E. coli* cells (15 h grown in LB broth and 20 h grown in LB + glycerol broth) were inoculated (5% v/v containing 10^6^ CFU/ml) into 100 ml of LB broth and 100 ml of LB + glycerol broth respectively and growth was monitored by measuring absorbance at 600 nm and the growth curves plotted (Figure 1).

### Growth of *E. coli* in Two Different Media for Gene Expression Studies

For gene expression studies three biological replicates were maintained for each medium (LB and LB+glycerol). Long-term stationary phase (LSP) cells (28 days old culture) incubated in both LB and LB + glycerol broth were transferred to fresh media and grown until 0.8 OD_600nm_ and used for gene expression studies. Similarly, stationary phase (SP) culture that is grown over night in LB broth (16 h) and LB + glycerol broth (20 h) were inoculated into fresh LB broth and LB + glycerol and grown till 0.8 OD_600nm_ and used for gene expression studies. After reaching 0.8 OD_600nm_, 10 ml of the culture was harvested and suspended in 3 volumes of RNA *later* solution (Ambion Inc., USA) and stored at 4°C. From the stored culture 3 ml was used for the isolation of RNA.

### RNA Extraction and cDNA Synthesis

Qiagen RNeasy mini-prep kit (Qiagen, USA) was used for the extraction of RNA from about 3 ml of *E. coli* culture and cDNA synthesized using the first strand cDNA synthesis kit from Invitrogen (Invitrogen Bioservices India Pvt. Ltd., Bangalore) as described previously [12]. The cDNA synthesized for all the three replicates of LSP and SP *E. coli* cultures grown freshly in both LB broth and LB+glycerol broth was fragmented with DNAse 1 (Promega Corporation, Madison, USA) and then labelled with biotin at the 3′ end using the labelling reagent from Affymetrix (CA, USA) and Terminal transferase enzyme (Promega Corporation, Madison, USA).

### DNA Microarray and Data Analysis

The labelled, fragmented cDNA of LSP and SP *E.coli* cultures was used to hybridize with the 20,366 genes representing four *E. coli* strains (viz., non-pathogenic *E. coli* K12 MG1655, uropathogenic *E. coli* strain CFT073 and enterohemorragic *E. coli* O157:H7 strains EDL 933 and Sakai). The *E. coli* gene chip arrays were purchased from Affymetrix and the hybridisation protocol followed was as per the Affymetrix protocol (www.affymetrix.com). The chip contained approximately 10,000 probe sets. Images of the microarray chips were scanned using Affymetrix 428 Array Scanner and GCOS software and subsequently processed to get intensity cell files for the probe sets. The intensity cell files obtained for all the replicates of LSP and SP *E. coli* cultures were then imported, normalized for background correction and data analysed using Gene Spring 11.5 software. Genes that exhibited ≥2.0 fold increase or decrease (treated versus control) in expression and P≤0.05 were considered as differentially regulated.

The microarray data was submitted to Gene Expression Omnibus (GEO) web deposit of National Centre for Biotechnology Information (NCBI) with an accession number GSE34275 for stationary phase grown *E. coli* cells and GSE50828 for long-term stationary phase *E. coli* cells grown in fresh medium.

### Real Time PCR

Validation of the differentially expressed genes was done as described previously [12]. The RT-PCR reactions (10 µl) were performed in triplicate for LSP and SP *E. coli* cells cDNA with, SYBR Green PCR Master Mix (Applied Biosystems, CA, USA) and 2.5 pM primer (Table 1). Template was pre-incubated at 50°C for 2 min, denatured at 95°C for 10 min and subjected to 40 cycles under the following thermal conditions: 95°C (15 s) and 52°C (30 s). Relative expression of genes (*insB, yhcE, ybfD, c3113, cysH, ydcC, oppA, csrC, dnaK, hdeA, ssrS, ryjA* and *aceB*) in LSP cultures grown in LB broth and eleven genes (*insB, yhcE, ybfD, c3113, cysH, ydcC, oppA, csrC, dnaK, hdeA* and *ssrS*) in LSP cultures grown in the presence of LB+glycerol was calculated by ΔΔC_T_ method which is based on product cycle threshold (C_T_). Expression of 16S rRNA gene was used as an internal standard for RT-PCR. All values reported represent the mean of at least three independent experiments.

**Table 1:** Primers used for Real-Time PCR analysis of a few genes of *E. coli*.

Sl.No.	Gene	Primer	Sequence (5′-3′)
1	*insB*	insB-rtF	GACTTTGTCATGCTGCTCCA
		insB-rtR	AGCGGCATAACCTGAATCTG
2	*yhcE*	yhcE-rtF	GGCCCGAATGGTTCATTAAA
		yhcE-rtR	TTGATGCTGTTCCTGTGCTG
3	*ybfD*	ybfD-rtF	TGCAGAAGGCTGGGAAGATA
		ybfD-rtR	TGGCAATGGTATCGTGAACA
4	*C3113*	C3113-rtF	GTTGTTCCGTGTCAGTGGTG
		C3113-rtR	TGCCTGAAAAATGAGCGAAC
5	*cysH*	cysH-rtF	CTGAATGCCAAAGCTGGAAG
		cysH-rtR	AACGCCGAACTGGAAAAACT
6	*ydcC*	ydcC-rtF	AATAAGCTGCACTGGCGTCT
		ydcC-rtR	ATGTGCCGTATCCCTGAAAA
7	*oppA*	oppB-rtF	GCGTATTACCCGTGGCTCTA
		oppB-rtR	TAATGCGTGGCGTAAAATGA
8	*csrC*	csrC-rtF	GAGGCGAAGACAGAGGATT
		csrC-rtR	CGTGTTGATTCCATTTCCGT
9	*dnaK*	dnaK-rtF	GGCACTGGCAACCGTACTAT
		dnaK-rtR	CCGTCAACTTCGTCGATTTC
10	*hdeA*	hdeA-rtF	CAGCCAGGAAATCTTCACAG
		hdeA-rtR	CTGCTTCTTCTGCCAGTTGT
11	*ssrS*	ssrS-rtF	CTCTGAGATGTTCGCAAGC
		ssrS-rtR	GTGTCGTCGCAGTTTTAAGG
12	*ryjA*	ryjA-rtF	CTTTCTCTCTATCCCGCTGGT
		ryjA-rtR	ACGTGCTCGAATGAGGTGTG
13	*aceB*	c4973-rtF	TTGCTGACTGTAGGCCGGAT
		c4973-rtR	TTCGGCAACGGCTGTAGG
14	*16S*	16S-rtF	GTGCAATATTCCCCACTGCT
		16S-rtR	CGATCCCTAGCTGGTCTGAG

### Assignment of the Differentially Regulated Genes to Functional Pathways by DAVID

Genes identified by microarray analysis were analyzed to identify relevant functional pathways by DAVID (Database for Annotation, Visualization and Integrated Discovery). A cutoff p value of 0.05 was used for enriched geneontology functions by DAVID. The genes that coded for unknown function were not included in the analysis.

## Results

### Effect of Long-term Stationary Phase on Growth of *E. coli* in LB Medium and in LB Medium Supplemented with Glycerol

*E. coli* culture that was stored for 28 days (LSP) showed decreased growth rate compared to stationary phase (SP) culture which was sub-cultured for growth (Figure 1) and the generation times were 153 and 45 min respectively. The presence of 10% glycerol in the medium further decreased the growth rate both in LSP culture and the SP culture and the generation times were 174 and 55 min respectively. Thus generation time of *E. coli* is increased due to LSP and presence of glycerol.

### Changes in Expression of Genes in LSP Cells of *E. coli* Cultured in LB Medium

**Figure 2:**
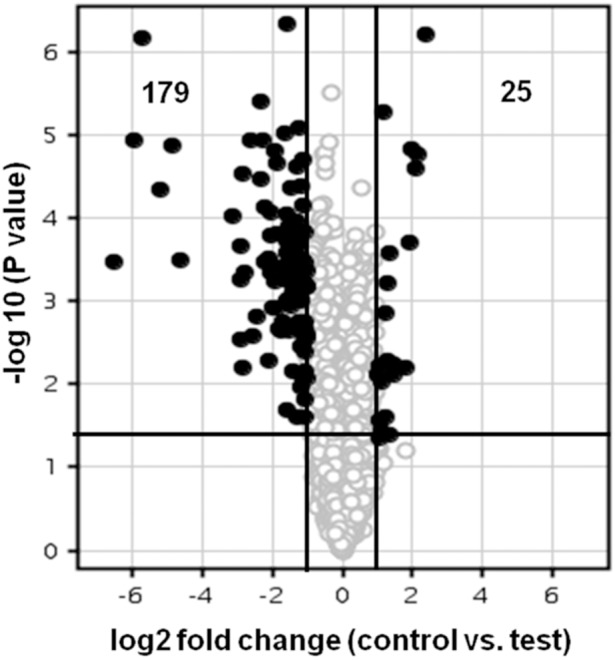
DNA microarray analysis of long-term stationary phase-induced gene expression in *E. coli* grown in LB broth. The Volcano plot depicts gene expression in 28 days old *E. coli* culture at 0.8 OD (OD_600nm_) cultured in LB broth compared to the freshly grown stationary phase *E. coli* culture control. Genes that are represented on the right side of the volcano-axis are up regulated and those that are on left side of the axis are down regulated. Out of the 4377 genes (O) analysed, 25 genes were up-regulated (•) and 179 were down regulated (•). Only those genes that showed more than 2.0 fold change in expression and a P value <0.05 were identified as either up- or down-regulated. The X-axis represents the log2 fold change and the dark vertical lines represent cut-offs at 2.0 fold decrease and increase. The y-axis represents the p-values and the dark horizontal line indicates a –log10 p value cut-off of 0.05.

Gene expression in *E. coli* that was stored for 28 days at room temperature (LSP) in LB broth and sub-cultured into a fresh medium was compared with stationary phase (SP) culture of *E. coli* which was sub-cultured for growth. Microarray analysis results indicated up regulation of twenty five genes and down regulation of one hundred and seventy nine genes in the LSP culture compared to the SP culture (Table S1 and S2 in File S1 and Figure 2). The up regulated genes included genes coding for transposase *(insB, two insC, insD, insE, two insF*, insH,and *insL*), for H repeat-containing protein (two *ydcC* and *ybfD*), for putative symporter (*yidK*), for nickel transporter subunit (*nikD*), for four hypothetical proteins (*c3113, c4174, c4965* and *ydiF*), for a pseudogene (*yhcE*) and six unknown genes (Table S1 in File S1).

The hundred and seventy nine down regulated genes comprised genes coding for metabolic pathways (29 genes), chaperons (3 genes), high pH media survival (9 genes), biofilm formation (1 gene), membrane components (5 genes) stationary phase survival proteins (5 genes), stress combating genes (6 genes) ribosomal protein synthesis (48 genes), DNA protecting (2 genes), non coding RNA genes (12 genes) hypothetical proteins (29 genes), other functional genes (22 genes) and unknown genes (9 genes) (Table S2 in File S1).

**Figure 3:**
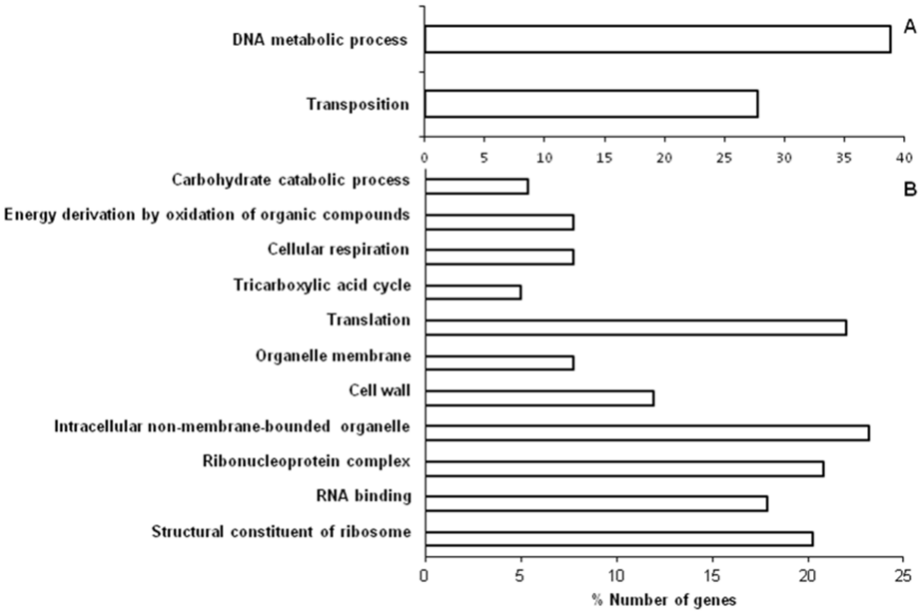
Genes up regulated (%) in *E. coli* LSP cells compared with SP cells of *E. coli* which were both sub-cultured in LB based on biological process classification reported by Gene ontology term functional categories using DAVID version 2.0 software (A). Genes down regulated (%) in *E. coli* LSP cells compared with SP cells of *E. coli* which were both sub-cultured in LB based on biological process classification reported by Gene ontology term functional categories using DAVID version 2.0 software (B).

DAVID was applied for the differentially expressed genes to get geneontology (GO) annotations and term enrichment categorisation for various biological processes (Figure 3A and 3B). GO terms enriched for more than 5% of genes are included in the figures. Based on DAVID, LSP *E. coli* cells grown in LB broth resulted in up regulation of two terms namely DNA metabolic process and transposition. While the down regulated genes resulted in enrichment of eleven terms, viz., carbohydrate catabolic process, energy derivation by oxidation of organic compounds, cellular respiration, tricarboxylic acid cycle, translation, organelle membrane, cell wall, intracellular non-membrane-bounded organelle, ribonucleoprotein complex, RNA binding and structural constituent of ribosome.

### Changes in Expression of Genes in LSP Cells of *E. coli* Cultured in LB Medium Supplemented with Glycerol

**Figure 4:**
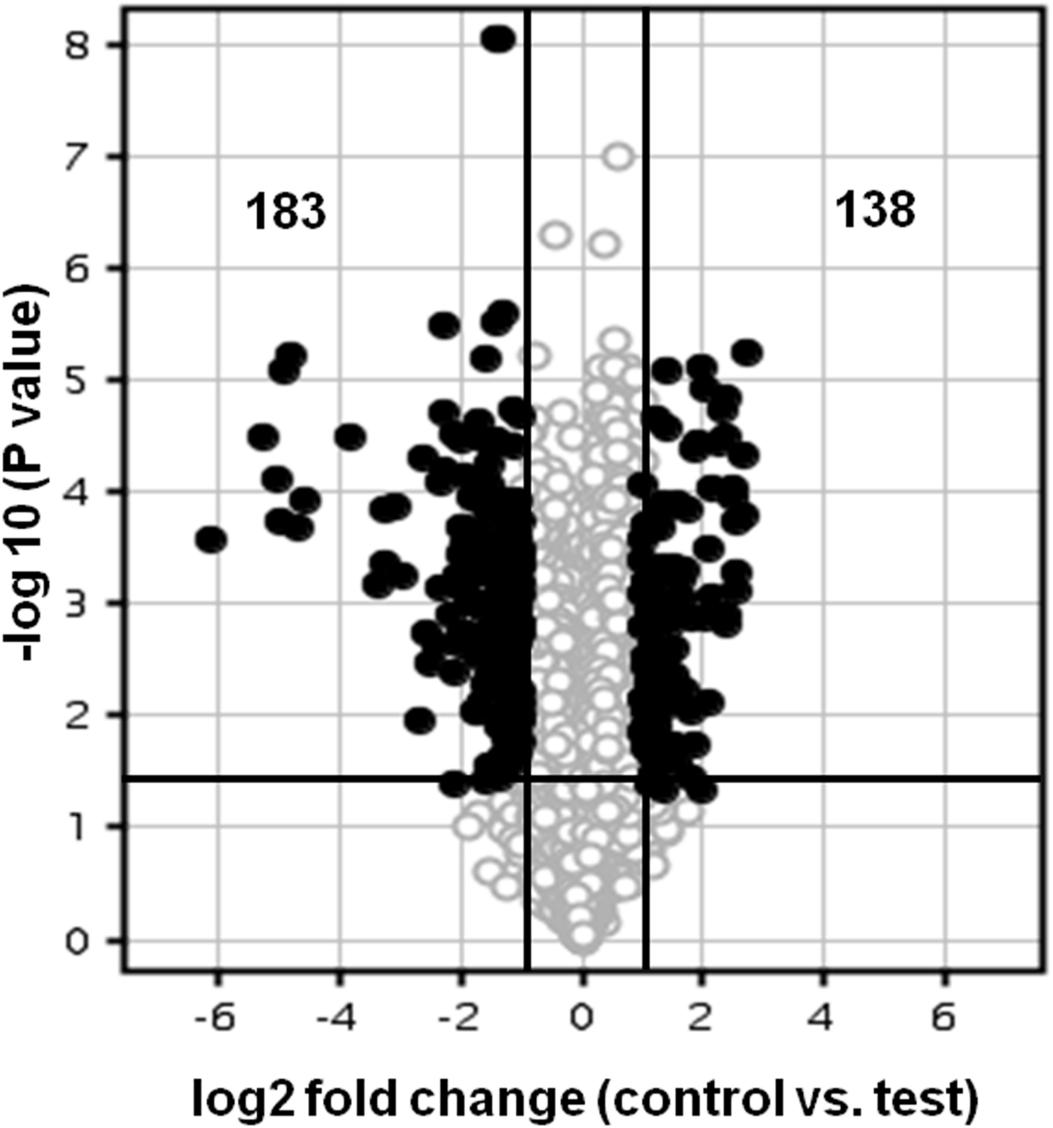
DNA microarray analysis of long-term stationary phase-induced gene expression in *E. coli* grown in LB broth plus glycerol. The Volcano plot depicts gene expression in LSP *E. coli* culture at 0.8 OD (OD_600nm_) cultured in LB broth compared to the freshly grown SP *E. coli* culture l. Genes that are represented on the right side of the volcano-axis are up-regulated and those that are on left side of the axis are down-regulated. Out of the 4377 genes (O) analysed, 138 genes were up-regulated (•) and 183 were down-regulated (•). Only those genes that showed more than 2.0 fold change in expression and a P value <0.05 were identified as either up- or down-regulated. The X-axis represents the log2 fold change and the dark vertical lines represent cut-offs at 2.0 fold decrease and increase. The y-axis represents the –log10 p-values and the dark horizontal line indicates a p value cut-off of 0.05.

In LSP culture of *E. coli* cultured in the presence of glycerol three hundred and twenty one genes (one hundred and thirty eight up-regulated and one hundred and eighty three down-regulated) were differentially regulated compared with overnight culture of *E. coli* which was cultured in the presence of glycerol (Table S3 and S4 in File S1 and Figure 4). The up-regulated genes included genes coding for transposases (7 genes), a pseudogene (1 gene), metabolic proteins (23 genes), other proteins (26 genes) hypothetical proteins (45 genes), non coding RNA (2 genes), pseudogenes (4 genes) and unknown genes (30 genes) (Table S3 in File S1).

The down regulated genes included genes coding for metabolic pathways (46 genes), high pH survival (6 genes), membrane components (6 genes), stationary phase survival (7 genes), other proteins (29 genes), ribosomal protein synthesis (11 genes), non coding RNA (10 genes), hypothetical proteins (59 genes) and eight unknown proteins (Table S4 in File S1).

**Figure 5:**
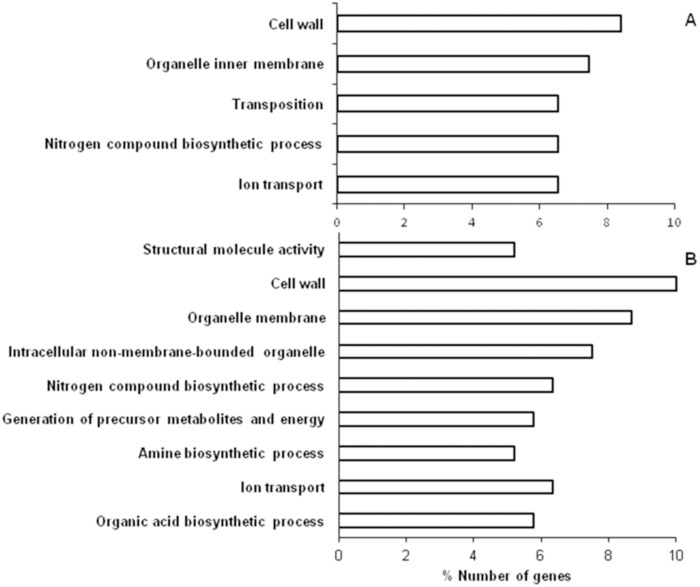
Genes up regulated (%) in *E. coli* LSP cells grown in LB + glycerol compared with stationary phase cells of *E. coli* which was sub-cultured in LB based on biological process classification reported by Gene ontology term functional categories using DAVID version 2.0 software (A). Genes down regulated (%) in *E. coli* LSP cells grown in LB + glycerol compared with stationary phase cells of *E. coli* which was sub-cultured in LB based on biological process classification reported by Gene ontology term functional categories using DAVID version 2.0 software (B).

Based on DAVID analysis LSP *E. coli* cells grown in LB broth supplemented with glycerol resulted in up-regulation of five gene ontology terms namely cell wall, organelle inner membrane, transposition, nitrogen compound biosynthetic process and ion transport (Figure 5A). Nine GO terms were enriched for the down-regulated genes, viz., structural molecule activity, cell wall, organelle membrane, intracellular non-membrane-bounded organelle, nitrogen compound biosynthetic process, generation of precursor metabolites and energy, amine biosynthetic process, ion transport and organic acid biosynthetic process (Figure 5B).

### Validation of the Expression of Genes by Real-Time PCR (RT-PCR)

**Figure 6:**
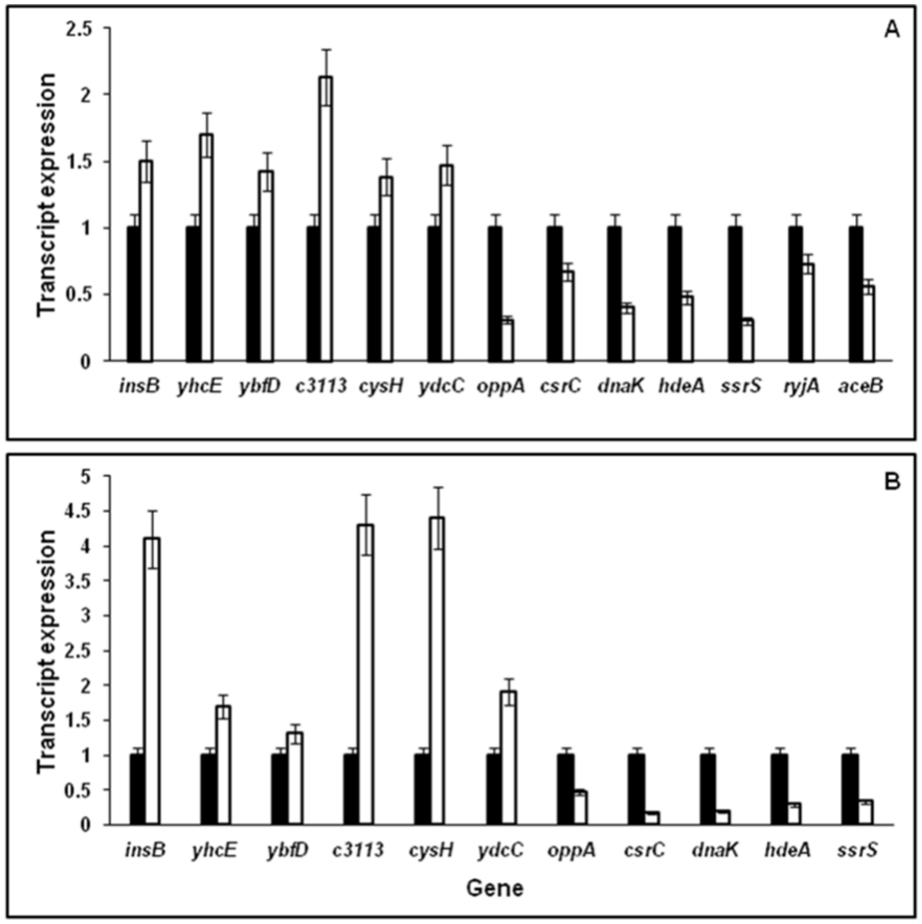
Real-Time PCR validation of the expression of *insB, yhcE, ybfD, c3113, cysH, ydcC, oppA, csrC, dnaK, hdeA, ssrS, ryjA* and *aceB* using RNA of LSP *E. coli* grown in LB broth (□) compared to stationary phase culture in LB broth (▪) (A). Real-Time PCR validation of the expression of *insB, yhcE, ybfD, c3113, cysH, ydcC, oppA, csrC, dnaK, hdeA, ssrS, ryjA* and *aceB* using RNA of LSP *E. coli* grown in LB+glycerol (□) compared to stationary phase culture grown in LB+glycerol (▪) (B).

Expression of thirteen genes (*insB, yhcE, ybfD, c3113, cysH, ydcC, oppA, csrC, dnaK, hdeA, ssrS, ryjA* and *aceB*) in LSP cultures grown in LB broth (Figure 6A) and eleven genes (*insB, yhcE, ybfD, c3113, cysH, ydcC, oppA, csrC, dnaK, hdeA and ssrS*) in LSP cultures grown in the presence of LB+glycerol (Figure 6B) were validated by RT-PCR and compared with the respective SP cells grown in the corresponding media. The expression of the genes was calculated based on the product Cycle threshold (C_T_) value. Statistical analysis of the data using ANOVA (Prism 3.0 software) indicated that the genes were either significantly up-or down regulated (P<0.05) in accordance with the DNA microarray results. The primers used for validation are given in Table 1.

## Discussion

Bacterial cells survive and live beyond stationary phase when kept for many days (long-term stationary phase) at room temperature. This adaptability is achieved by changes in cell envelope [13], metabolism [1] and the concurrent regulation of genes. With this in view, in the present study, effect of long-term stationary phase on growth and global gene expression was monitored in *E. coli* using DNA microarray analysis.

Both the stationary phase sub-cultured cells and LSP sub-cultured cells exhibited similar growth patterns but in LSP cells the lag phase is delayed and the growth rate is retarded (Figure 1). Pin and Baranyi, [14] had suggested that the length of the lag phase varies depending on the bacterial species, the new environment and importantly the length of time that the cells have starved prior to the new environment created due to sub culturing in to a fresh medium [14]. Thus it is possible that since the LSP cells were retained in the stationary phase for 28 days prior to sub culturing (the new environment) they required a longer period to adjust to the changed media conditions compared to the stationary phase cells. Subsequently both the LSP and SP cells entered the exponential phase of growth but the LSP cells exhibited a longer generation time (153 min) compared to the SP cells (45 min). The medium used for sub culturing was LB therefore the slow generation time in LSP may not be attributed to the nutrient status of the medium as indicated earlier that the generation time is slower in nutrient-poor conditions compared to nutrient-rich conditions [2]. We are not aware of similar comparisons in growth characteristics of SP cells and LSP sub-cultured cells in literature.

Several studies have indicated that *E. coli* in long-term stationary phase for 10 days yield cells that express the growth advantage in stationary phase (GASP) phenotype due to a mutation in *rpoS*, which encodes a putative stationary phase-specific sigma factor [3], [15]–[17]. These cells with GASP phenotype (LSP) when mixed with fresh cultures either transiently or permanently- outnumber the younger population with time or aged cells are unable to compete with younger strains [1]. In these earlier experiments LSP and fresh cells were mixed to ascertain the advantage if any due to GASP by monitoring the number of viable cells (Colony forming units) and not the growth characteristics as in the present study. Thus the two experiments should not be strictly compared but what could be deduced is that at least the LSP cells did not exhibit any growth advantage for a short period of the experiment (24 h). Presence of glycerol in the LB medium further decreased the growth rate in both the SP sub-cultured cells and LSP sub-cultured cells.

Previous studies have indicated that gene expression is influenced by growth rate [18]–[20], growth rate-dependent gene copy number, abundance of RNA polymerase and ribosomes [21]. Further, Klump et al., [11] demonstrated that mRNA abundance is a good candidate for measurement of gene expression and it does not show growth rate dependence. Hence, in the present study, to assess global gene expression changes occurring due to LSP, DNA microarray analysis was compared between SP sub-cultured cells and LSP grown cells that were cultured in two different media, LB and LB supplemented with glycerol (10% v/v).

### Changes in Expression of Genes in *E. coli* due to Long-term Stationary Phase Effect in LB Medium

LSP *E. coli* cells when grown in LB broth resulted in differential expression of 204 genes (25 genes were up-regulated and 179 genes were down regulated) (Table S1 and S2 in File S1 and Figure 2). None of the up-regulated genes coded for genes associated with stress. At the same time, as anticipated, it was observed that the down-regulated genes comprised genes which would prolong the lag phase and increase the generation time like genes pertaining to metabolic pathways (29 genes), ribosomal protein synthesis (47), DNA protecting (2), genes required for high pH media survival (9), stationary phase survival proteins (5), stress combating genes (6), chaperon functional activity (3), biofilm formation (1) and membrane components (5). It was also observed that a significant number of the up and down regulated genes were categorised as hypothetical protein coding genes and unknown genes (Table S1 and S2 in File S1). DAVID gene ontology (GO) term enrichments for LSP cells grown in LB broth also confirmed down regulation in enrichments for carbohydrate catabolic process, energy derivation by oxidation of organic compounds, cellular respiration, tricarboxylic acid cycle (corresponding to metabolic pathway genes), translation, ribonucleoprotein complex, RNA binding and structural constituent of ribosome (corresponding to ribosomal protein synthesis genes), organelle membrane, cell wall, intracellular non-membrane-bounded organelle (corresponding to genes coding for membrane components) (Figure 3B) thus providing justification for the observed lag phase delay and slower growth rate in LSP cells.

### Changes in Expression of Genes in *E. coli* due to Long-term Stationary Phase Effect in LB Medium Supplemented with Glycerol

LSP *E. coli* cells when grown in LB broth supplemented with glycerol (10% v/v) resulted in differential expression of 321 genes (138 genes were up-regulated and 183 genes were down regulated) (Table S3 and S4 in File S1). The presence of glycerol in the LB medium appeared to up regulate genes coding for metabolic proteins in addition to the genes coding for transposases, hypothetical proteins, a pseudogene and unknown genes as in LSP cells grown in the absence of glycerol. This up-regulation of metabolic genes was not sufficient to restore the growth phenotype observed in SP sub-cultured cells. Presence of glycerol also down regulated the genes that were down regulated in the absence of glycerol in the LSP cells except it did not down regulate genes coding for stress genes, DNA protection and chaperones. Nevertheless the growth phenotype indicated a prolonged lag phase and decreased generation time. DAVID analysis of LSP *E. coli* cells grown in LB broth supplemented with glycerol resulted in down-regulation of gene ontology terms such as nitrogen compound biosynthetic process, generation of precursor metabolites and energy, amine biosynthetic process, ion transport and organic acid biosynthetic process which may adversely affect the growth as observed.

### Effect of Glycerol on Expression of Genes in LSP Cells of *E. coli*

In our previous investigations it was observed that *E. coli* cells when grown in LB broth, the pH of the medium increased from 7 to 8.2 when the cells reached the stationary phase. But when the cells were grown in LB broth supplemented with glycerol the pH of the medium in the stationary phase decreased to 4.6. Thus growth rate and media pH are the other parameters that would influence the LSP cells.

A more detailed discussion on the genes which are up- or down-regulated due to LSP irrespective of whether the medium is LB or LB supplemented with glycerol is given and interpreted with respect to the possible role of these genes in influencing the growth phenpotype. A total of 95 genes showed significantly up or down regulation due to LSP effect irrespective of the medium used (LB or LB plus glycerol) (Table 2 and 3). The 22 up regulated genes included genes coding for insertion sequences *(insB, two insC, insD, insE, two insF*, *insH,* and *insL*), for H repeat-containing protein (two *ydcC* and *ybfD*), for putative symporter (*yidK*), for nickel transporter subunit (*nikD*), for three hypothetical proteins (*c3113, c4174* and *ydiF*), for a pseudogene (*yhcE*) and four unknown genes (Table 2). The 73 down regulated genes included, genes coding for stress and stationary phase response (9 genes), ribosomal protein synthesis and related genes (14 genes), membrane bound and transport related genes (6 genes), non coding RNA genes (11 genes), hypothetical proteins (16 genes), metabolic other functional genes (12 genes*)* and unknown genes (5 genes) (Table 3).

**Table 2:** List of up-regulated genes in *Escherichia coli* due to long-term stationary phase effect.

Sl. No	Probe set ID		Gene	Gene function
Genes coding for insertion sequences				
1		1762495_s_at	*insB*	IS1 transposase InsAB’
2		1761145_s_at	*insC*	KpLE2 phage-like element; IS2 insertion element repressor InsA
3		1766261_x_at	*insC*	KpLE2 phage-like element; IS2 insertion element repressor InsA
4		1759674_s_at	*insD*	insertion element IS2 transposase InsD
5		1766923_s_at	*insE*	IS3 element protein InsE
6		1764530_s_at	*insF*	IS3 element protein InsF
7		1766257_s_at	*insF*	IS3 element protein InsF
8		1765954_s_at	*insH*	IS5 transposase and trans-activator
9		1768300_s_at	*insL*	IS186/IS421 transposase
Other up-regulated genes				
10	1766930_s_at		*ydcC*	H repeat-containing protein
11	1767281_s_at		*ydcC*	H repeat-containing protein
12	1763291_x_at		*ybfD*	H repeat-containing protein
13	1765969_s_at		*yidK*	putative symporter YidK
14	1759669_s_at		*nikD*	nickel transporter subunit
15	1766017_s_at		*ydiF*	fused predicted acetyl-CoA:acetoacetyl-CoA transferase: alpha subunit/beta subunit
16	1767447_s_at		*yhcE*	Pseudogene
17	1768337_s_at		*c3113*	hypothetical protein
18	1761612_s_at		*c4174*	hypothetical protein
19	1762957_s_at		-	Unknown
20	1765228_s_at		-	Unknown
21	1764872_s_at		-	Unknown
22	1766610_s_at		-	Unknown

Genes that showed fold change greater than 2.0 (P<0.05).

**Table 3:** List of down-regulated genes in *Escherichia coli* due to long-term stationary phase effect.

Sl. No	Probe set ID	Gene		Gene function
Genes coding for stress and stationary phase response				
1	1766431_s_at		*argH*	argininosuccinate lyase
2	1764240_s_at		*talA*	transaldolase A
3	1765284_s_at		*raiA*	translation inhibitor protein RaiA
4	1759918_s_at		*clpA*	ATPase and specificity subunit of ClpA-ClpP ATP-dependent serine protease, chaperone activity
5	1761735_s_at		*wrbA*	TrpR binding protein WrbA
6	1765321_s_at		*hdeA*	stress response protein acid-resistance protein
7	1761492_s_at		*ihfA*	integration host factor subunit alpha
8	1760008_s_at		*rmf*	ribosome modulation factor
9	1768544_at		*papD_2*	PapD protein
Ribosomal protein synthesis and related genes				
10	1761999_s_at		*fusA*	elongation factor G
11	1768334_s_at		*infC*	translation initiation factor IF-3
12	1767807_s_at		*rpmF*	50S ribosomal protein L32
13	1765214_s_at		*rpsB*	30S ribosomal protein S2
14	1760241_s_at		*rpsF*	30S ribosomal subunit protein S6
15	1760745_s_at		*rpsL*	30S ribosomal subunit protein S12
16	1763008_s_at		*rpsN*	30S ribosomal protein S14
17	1767590_s_at		*rpsV*	30S ribosomal subunit S22
18	1763891_s_at		*rrfA, rrfB, rrfC, rrfD, rrfE, rrfF, rrfG, rrfH*	5S ribosomal RNA of rrnA, rrnB, rrnC, rrnD, rrnE, rrnG, rrnH operon
19	1760713_s_at		*rrlA, rrlB, rrlC, rrlC, rrlD, rrlE, rrlG, rrlH*	23S ribosomal RNA of rrnA rrnB, rrnC, rrnD, rrnE, rrnG, rrnH operon
20	1766157_s_at		*rrsA, rrsB, rrsC, rrsD, rrsE, rrsG*	16S ribosomal RNA of rrnA, rrnB, rrnC, rrnD, rrnE, rrnG, operon
21	1763456_s_at		*rrsA, rrsB, rrsC, rrsD, rrsE, rrsG rrsH*	16S ribosomal RNA of rrnH operon
22	1767883_s_at		*rimM*	16S rRNA-processing protein RimM
23	1767263_s_at		*eno*	phosphopyruvate hydratase
Genes coding for transporters				
24	1765595_s_at	*ompA*		outer membrane protein A
25	1768937_at	*ompC*		outer membrane porin protein C
26	1759780_s_at	*lpp*		murein lipoprotein
27	1764969_s_at	*oppA*		oligopeptide transport periplasmic binding protein
28	1765506_s_at	*livM*		leucine/isoleucine/valine transporter permease subunit
29	1761034_at	*aefA*		potassium efflux protein KefA
Non coding RNA genes				
30	1763312_at	*csrB*		ncRNA
31	1768835_at	*csrC*		ncRNA
32	1766397_s_at	*csrC*		ncRNA
33	1762278_at	*rybA*		ncRNA
34	1760679_at	*ryjA*		ncRNA
35	1763985_s_at	*ffs*		misc_RNA
36	1762699_at	*micF*		misc_RNA
37	1767680_at	*gcvB*		ncRNA
38	1762568_s_at	*ssrA*		misc_RNA
39	1763089_s_at	*ssrS*		ncRNA
40	1760716_s_at	*rnpB*		ncRNA
Other functional genes				
41	1765424_at	*nikE*		nickel transporter ATP-binding protein NikE
42	1766199_at	*agaS*		putative tagatose-6-phosphate ketose/aldose isomerise
43	1764443_s_at	*gatB*		galactitol-specific PTS system component IIB
44	1760076_at	*glpC*		sn-glycerol-3-phosphate dehydrogenase subunit C
45	1761419_s_at	*speB*		Agmatinase
46	1769115_s_at	*fabG*		3-ketoacyl-(acyl-carrier-protein) reductase
47	1767961_s_at	*ahpC*		alkyl hydroperoxide reductasesubunit C
48	1760565_at	*c0336*		PTS system, mannitol (Cryptic)-specific IIA component
49	1759877_at	*c3406*		phosphosugar isomerise
50	1764975_s_at	*ycjU*		putative beta-phosphoglucomutase
51	1768861_s_at	*ECs1203*		antitermination protein Q
52	1763881_s_at	*cydA*		cytochrome d terminal oxidase, polypeptide subunit I
Hypothetical/unknown functional genes				
53	1762658_s_at	*yeaG*		hypothetical protein
54	1764346_at	*ECs4566*		hypothetical protein
55	1767843_s_at	*ECs0302*		hypothetical protein
56	1766612_at	*c0339*		hypothetical protein
57	1761825_at	*c1191*		hypothetical protein
58	1761672_at	*c2430*		hypothetical protein
59	1765596_at	*c2814*		hypothetical protein
60	1768188_at	*c3657*		hypothetical protein
61	1768540_at	*c4419*		hypothetical protein
62	1768600_s_at	*c5154*		hypothetical protein
63	1764543_at	*chuY*		hypothetical protein
64	1763144_at	*yedK*		hypothetical protein
65	1765109_at	*yggR*		hypothetical protein
66	1767797_at	*yhjX*		hypothetical protein
67	1768987_at	*yjeF*		hypothetical protein
68	1768179_at	*Z5095*		hypothetical protein
69	1760017_at	*-*		Unknown
70	1759110_s_at	*-*		Unknown
71	1765325_s_at	*-*		Unknown
72	1762413_s_at	*-*		Unknown
73	1764615_s_at	*-*		Unknown

Genes that showed fold change greater than 2.0 (P<0.05).

### Genes Coding for Insertion Sequences

Out of the eight different insertion sequence elements that are present in *E. coli* K-12, genes belonging to four of them IS1, IS2, IS3 and IS5 were up regulated due to LSP effect in *E. coli*. This may be attributed to the physiological status of the cell [22] and to the fact that such insertion sequences confer beneficial effects during starvation than during growth [23].

### Other Up-regulated Genes in LSP Cells

*ydcC* and *ybfD* code for H repeat-containing proteins. The functions of these two genes is not known but both are putative transposases. The YidK protein encoded by *yidK* is a sodium dependent solute transporter and may function as a sodium-driven metabolite uptake system. Gene *yhcE* is a pseudogene which codes for an uncharacterised protein YhcE. Pseudogenes are disabled copies of functional genes but are known to exhibit differential expression [24].

### Genes Related to Stress and Stationary Phase Response

A total of 9 genes coding for stress and stationary phase response are down regulated in LSP cells. Two of these genes *argH* which codes for argininosuccinate lyase function [25] and *talA* which codes for transaldolase, a key enzyme in the non-oxidative pentose phosphate pathway [26] are regulated by the general stress regulator sigmaS (or RpoS) subunit of RNA polymerase. The down-regulation of *argH* and *talA* may be due to the down regulation of Rpos gene which was seen in LSP cells (Table 3). The end products of *argH* and *talA* genes are also important for optimum growth of the cells.

The other stress induced genes that are down-regulated include *raiA* coding for translation inhibitor protein RaiA and *rmf* coding for ribosome modulator factor gene which are required for translation and thus down-regulation would affect growth during starvation. RaiA is a stress induced protein produced during stationary phase [27] and the other protein ribosomal modulator factor (*rmf*) expression is influenced by the growth phase and is inversely related to the growth rate [26]. *rmf* gene may be involved in modulating the ribosomes to form dimers during stationary phase for the long term survival of cells [28]. These dimers would be converted to 70S ribosomes upon transferring of stationary phase cells to fresh medium [29], [30], thus helping in resuming the normal translational process. It was experimentally demonstrated that *rmf* was silent in rapidly growing exponential phase cells but high level of transcription occurs concomitantly with the growth transition to stationary phase [28]–[30].

Gene *clpA* codes for ClpATPases that are implicated in the stress tolerance of many microorganisms [31], [32] since they perform the chaperon function of DnaK and GroEL under stress [33].

The gene *wrbA* codes for the tryptophan repressor- binding protein WrbA and this protein is a stationary phase induced protein. But the gene was down regulated in LSP cells. It has been proposed that the WrbA protein functions as an accessory element in blocking tryptophan repressor-specific transcriptional processes [34].

Gene *ihfA* is down regulated in LSP cells and may thus be responsible for the growth phenotype of LSP cells because this gene encodes for integration host factor, which is a global regulatory protein that helps to maintain DNA architecture by influencing DNA supercoiling, DNA duplex destabilization, DNA replication, recombination and the expression of many genes [35]–[37].

Gene *papD2* encodes for PapD protein that functions as putative chaperone-usher fimbrial operon in *E. coli* K12 [38]. Transcription of PapD is regulated by gene *ihfA* which is also down-regulated in LSP cells.

HdeA is the most abundant protein in the periplasmic space of *E. coli* during stationary phase when grown in a nutrient rich media [39] thus implying its importance in stationary phase of growth. The down-regulation of *hdeA* gene observed in LSP cells would thus affect the growth.

All these stress and stationary phase response genes showed down-regulation in LSP cells compared to SP cells; however from literature it is apparent that these genes are also down regulated during stationary phase [25]–[39]. Therefore, the observed down regulation in majority of the stress and stationary phase response genes could be attributed both to stationary phase and LSP. In addition these results imply that during re-growth of LSP and SP cells the transcripts for stress and stationary phase response genes are more lowered in LSP cells compared to SP cells and this could be the reason for the observed retardation of growth in LSP cells compared to SP cells.

### Genes Coding for Ribosomal Protein Synthesis and Related Genes

The gene encoding for translation initiation factor IF-3, gene *fusA,* some of the 50S and 30S ribosomal protein synthesizing genes and *rrn* operons for 5S, 16S and 23S ribosomal RNA were down-regulated in LSP cells than in SP cells. Other genes that belong to this group of down-regulated genes include *infC* which codes for the protein, translation initiation factor IF-3, plays a crucial role in functional interaction of mRNA with 30S ribosome [40], gene *fusA* that encodes for elongation factor G which is an essential protein that facilitates the translocation of the ribosome by one codon along the mRNA molecule [41]. Further, genes coding for 30S ribosomal proteins (*rpsB, rpsF, rpsL, rpsN* and *rpsV*) and 50S ribosomal proteins (*rpmF*) were also down regulated. Thus compared to the SP cells, in LSP cells genes required for initiation and elongation of translation and genes coding for ribosomal proteins are down regulated thus affecting the growth of LSP cells. Further, redundancy in *rrn* operon, as seen in LSP cells reduces the ribosomal number in the cell, thus decreasing cell proliferation [42]. Earlier studies have shown that deletion of the *trmD* gene or *rimM* resulted in five to seven folds reduction in the growth of the cells [43]. The low abundance of these transcripts also contributes to the slow growth rate in LSP cells of *E. coli*. Gene *eno* encoding for phosphopyruvate hydratase is a component of RNA degradosome and plays a vital role in RNA processing and messenger RNA degradation [44]. The down-regulation of this degradosome may be an adaptive strategy employed by LSP cells to facilitate the growth of the cells.

### Genes Coding for Transporters

Several genes coding for transport related proteins such as OmpC, OmpA, Lpp, OppA, LivM and AefA were down-regulated. As a consequence the diffusion of small hydrophilic molecules across the membrane [45] would be affected. It is also known that in *E. coli* the expression of porins OmpF and OmpC are regulated under certain stress conditions including changes in osmotic pressure, temperature and pH [45]. This study indicates down regulation of these genes due to long-term stationary phase. Earlier studies have also indicated that *ompC* gene expression is regulated depending on the pH conditions of the extracellular medium [46]–[48]. Thomas and Booth [46] also demonstrated under acidic pH conditions *ompC* gene regulation depends on the carbon source used in the medium with glycerol repressing the expression of *ompC* as observed in the present study. However, it was reported that the expression of *ompA* is decreased due to sigma E factor envelope stress response [49] indicating its role in response to long-term stationary phase effect. For the optimum growth and functioning, the cell requires various other transport proteins like the permeases that help the cell in regulating cell volume [50] by adjusting the ion and water transport across the cell membrane [51], and thereby protecting the DNA and protein structure. These permeases include the oligopeptide transporter permease coding gene *oppA* which mediates uptake of both dipeptides and oligopeptides [52], the *aefA* gene that codes for potassium efflux protein KefA which functions as a mechano-sensitive channel [53] and the gene *livM* that codes for branched chain aminoacid (leucin/isoleucine/valine) transporter permeases [54]. Down regulation of these proteins would have a negative effect on the growth of the cells as observed in LSP cells. In the present situation, since the general stress regulator is showing down regulation in its gene expression, thus, the transcripts pertaining to certain permeases have showed low abundance.

### Non-coding RNA Genes

Several genes coding for non-coding RNA like *csrB and csrC* genes showed decreased expression in LSP cells. This was anticipated since earlier studies had indicated that in *E. coli csrB* and *csrC* genes were down regulated in medium like LB which contains casaminoacids [55]. The other non coding RNA gene *ssrS* was also down regulated in LSP cells. SsrS plays an important role in sigma 70 dependant transcription [56], [57] and is thus important for growth. The other non-coding RNA genes which were down regulated are also important since they are involved in varies functions such as *rybA* which acts as a manganese chaperon [58], *ffs* gene which is essential for protein synthesis [59], GcvB which is a small regulatory RNA involved in the regulation of amino acid availability [60] and *ryjA* gene which codes for novel small RNA with an unknown function.

### Genes Coding for Nickel Transporter Subunit (*nikD*), a Number of Hypothetical Genes and Unknown Genes are Both Up or Down Regulated in LSP

In the present study two genes *nikD* and *nikE* that encode for the NikABCDE ATP-dependent nickel (II) uptake system are contradictingly differentially regulated. Gene *nikD* has shown up-regulation while gene *nikE* is down-regulated in LSP cells. The reason for this is difficult to explain but it is known that these genes code for ATP-dependent nickel (II) uptake which may be needed for a variety of enzymatic reactions in prokaryotes. It is also known that high intracellular concentrations of nickel are toxic since they catalyze the formation of reactive forms of oxygen that can damage cellular constituents.

In addition it has also been observed that several genes coding for hypothetical proteins and genes whose functions are unknown are either up-or down- regulated in the LSP cells and only functional characterisation of these genes would shed light on their role with respect to LSP effect on cells (Table 3).

### Others Differentially Down-regulated Genes

It is indeed not surprising to find that several metabolic genes are down regulated and these include *agaS* (that codes for putative tagatose-6-phosphate ketose/aldose isomerise required for galactosamine utilization) [61], gene *gatB* (that codes for galactitol-specific PTS system component IIB) and gene *glpC* (that codes for anaerobic glycerol 3-phosphate dehydrogenase). *glpC* expression is influenced by *dps* [62] gene, which codes for a non specific DNA-binding protein which is abundant during stationary phase and protects the cells from oxidative stress, UV and gamma irradiation, iron and copper toxicity, thermal stress and pH stress. The other metabolic genes that are down-regualted in LSP cells include *speB* gene, which codes for the putrescine-biosynthetic enzyme agmatinase [63] and *fabG* gene coding for 3-oxoacyl-[acyl-carrier-protein] reductase which is involved in fatty acid synthesis [64].

## Conclusions

The study confirms and reiterates the importance of genes involved in stress, ribosomal protein synthesis and transport as crucial for growth in LSP cells. It also provides evidence for a positive role for insertion sequences and transposaes in LSP cells. Down-regulation of gene *ihfA* a global regulatory protein that helps to maintain DNA architecture and the expression of many genes is a very important observation which would help to understand the molecular basis of retarded growth in LSP cells. Further down-regulation of genes involved in degradosome may be an adaptive strategy employed by LSP cells to facilitate the growth of the cells. An interesting observation of the study is the differentially regulation of several genes coding for hypothetical proteins, pseudogenes and unknown genes which need to be characterised to get a better insight into the molecular basis of survival and growth of LSP cells.

## Supporting Information

File S1 **Supplementary tables. Table S1. Up-regulation of genes in** ***Escherichia coli*** **long-term stationary phase cells in LB medium.** DNA microarray analysis of LSP *E. coli* cells grown in the presence of LB broth showed up regulation of 25 genes with a fold change >2.0 (P<0.05). **Table S2. Down-regulation of genes in** ***Escherichia coli*** **long-term stationary phase cells in LB medium.** DNA microarray analysis of LSP *E. coli* cells grown in the presence of LB broth showed down regulation of 179 genes with a fold change >2.0 (P<0.05). **Table S3. Up-regulation of genes in** ***Escherichia coli*** **long-term stationary phase cells in LB medium supplemented with glycerol.** DNA microarray analysis of LSP *E. coli* cells grown in the presence of LB broth supplemented with glycerol showed up regulation of 138 genes with a fold change >2.0 (P<0.05). **Table S4. Down-regulation of genes in** ***Escherichia coli*** **long-term stationary phase cells in LB medium supplemented with glycerol.** DNA microarray analysis of LSP *E. coli* cells grown in the presence of LB broth supplemented with glycerol showed down regulation of 183 genes with a fold change >2.0 (P<0.05).
(DOCX)
