# Supplementary material for: Global Gene Expression Analysis of Long-Term Stationary Phase Effects in E. coli K12 MG1655
Source: PLoS One. 2014 May 23;9(5):e96701. doi: 10.1371/journal.pone.0096701 (PMC4032248; doi:10.1371/journal.pone.0096701)
Supplement: File S1 — Supplementary tables. Table S1. Up-regulation of genes in Escherichia coli long-term stationary phase cells in LB medium. DNA microarray analysis of LSP E. coli cells grown in the presence of LB broth showed up regulation of 25 genes with a fold change >2.0 (P<0.05). Table S2. Down-regulation of genes in Escherichia coli long-term stationary phase cells in LB medium. DNA microarray analysis of LSP E. coli cells grown in the presence of LB broth showed down regulation of 179 genes with a fold change >2.0 (P<0.05). Table S3. Up-regulation of genes in Escherichia coli long-term stationary phase cells in LB medium supplemented with glycerol. DNA microarray analysis of LSP E. coli cells grown in the presence of LB broth supplemented with glycerol showed up regulation of 138 genes with a fold change >2.0 (P<0.05). Table S4. Down-regulation of genes in Escherichia coli long-term stationary phase cells in LB medium supplemented with glycerol. DNA microarray analysis of LSP E. coli cells grown in the presence of LB broth supplemented with glycerol showed down regulation of 183 genes with a fold change >2.0 (P<0.05). (DOCX) [file pone.0096701.s001.docx]

**Table S1.** Up regulation of genes in *Escherichia coli* long-term stationary phase cells in LB medium

| **Sl. No** | **Probe set ID** | **Fold change** | **Gene** | **Gene function** |
| --- | --- | --- | --- | --- |
| 1 | 1762495_s_at | 4.0 | *insB* | IS1 transposase InsAB' |
| 2 | 1761145_s_at | 3.3 | *insC* | IS2 insertion element repressor InsA |
| 3 | 1766261_x_at | 2.2 | *insC* | IS2 insertion element repressor InsA |
| 4 | 1759674_s_at | 5.3 | *insD* | insertion element IS2 transposase InsD |
| 5 | 1766923_s_at | 2.3 | *insE* | IS3 element protein InsE |
| 6 | 1764530_s_at | 2.6 | *insF* | IS3 element protein InsF |
| 7 | 1766257_s_at | 4.5 | *insF* | transposase |
| 8 | 1765954_s_at | 4.2 | *insH* | IS5 transposase and trans-activator |
| 9 | 1768300_s_at | 2.8 | *insL* | IS186/IS421 transposase |
| 10 | 1766930_s_at | 3.8 | *ydcC* | H repeat-containing protein |
| 11 | 1767281_s_at | 2.1 | *ydcC* | H repeat-containing protein |
| 12 | 1763291_x_at | 3.3 | *ybfD* | H repeat-containing protein |
| 13 | 1765969_s_at | 2.1 | *yidK* | putative symporter YidK |
| 14 | 1759669_s_at | 2.0 | *nikD* | nickel transporter subunit |
| 15 | 1767447_s_at | 5.0 | *yhcE* | pseudo |
| 16 | 1768337_s_at | 4.6 | *c3113* | hypothetical protein |
| 17 | 1761612_s_at | 2.9 | *c4174* | hypothetical protein |
| 18 | 1768701_at | 2.3 | *c4965* | hypothetical protein |
| 19 | 1766017_s_at | 2.3 | *ydiF* | hypothetical protein |
| 20 | 1762957_s_at | 2.2 |  |  |
| 21 | 1765228_s_at | 3.0 |  |  |
| 22 | 1764872_s_at | 2.5 |  |  |
| 23 | 1764616_s_at | 2.5 |  |  |
| 24 | 1761930_s_at | 2.2 |  |  |
| 25 | 1766610_s_at | 2.7 |  |  |

Genes that showed fold change greater than 2.0 (P < 0.05)

**Table S2.** Down regulation of genes in *Escherichia coli* long-term stationary phase cells in LB medium

| **Sl. No** | **Probe set ID** | **Fold change** | **Gene** | **Gene function** |
| --- | --- | --- | --- | --- |
| 1 | 1763981_s_at | 6.2 | *aceA* | isocitrate lyase |
| 2 | 1761179_at | 3.7 | *aceB* | malate synthase |
| 3 | 1762343_s_at | 3.8 | *aceB* | malate synthase |
| 4 | 1762781_at | 3.2 | *aceK* | isocitrate dehydrogenase kinase/phosphatase |
| 5 | 1765758_s_at | 2.4 | *aceK* | bifunctional isocitrate dehydrogenase kinase/phosphatase protein |
| 6 | 1766568_s_at | 3.0 | *acpP* | acyl carrier protein |
| 7 | 1762651_s_at | 3.0 | *adk* | adenylate kinase |
| 8 | 1761034_at | 2.3 | *aefA* | potassium efflux protein KefA |
| 9 | 1766199_at | 2.5 | *agaS* | putative tagatose-6-phosphate ketose/aldose isomerase |
| 10 | 1767961_s_at | 2.3 | *ahpC* | alkyl hydroperoxide reductase subunit C |
| 11 | 1760980_s_at | 2.0 | *alpA* | prophage CP4-57 regulatory protein alpA |
| 12 | 1768341_s_at | 2.4 | *aspA* | aspartate ammonia-lyase |
| 13 | 1760565_at | 3.2 | *c0336* | PTS system, mannitol (Cryptic)-specific IIA component |
| 14 | 1768731_at | 2.2 | *c1175* | putative aminotransferase |
| 15 | 1761030_s_at | 2.0 | *rpoS* | RNA polymerase, sigma S (sigma 38) factor |
| 16 | 1759877_at | 2.6 | *c3406* | phosphosugar isomerase |
| 17 | 1764713_s_at | 3.1 | *yiaG* | predicted transcriptional regulator |
| 18 | 1759918_s_at | 2.1 | *clpA* | ATP-dependent Clp protease ATP-binding subunit |
| 19 | 1763709_s_at | 2.1 | *clpP* | ATP-dependent Clp protease proteolytic subunit |
| 20 | 1761682_s_at | 2.4 | *cpxP* | periplasmic protein combats stress |
| 21 | 1767310_s_at | 2.5 | *crp* | DNA-binding transcriptional dual regulator |
| 22 | 1766690_s_at | 6.1 | *cspE* | cold shock protein CspE |
| 23 | 1763881_s_at | 3.6 | *cydA* | cytochrome d terminal oxidase, polypeptide subunit I |
| 24 | 1769018_s_at | 2.6 | *cyoA* | cytochrome o ubiquinol oxidase subunit II |
| 25 | 1763357_s_at | 5.8 | *dnaK* | molecular chaperone DnaK |
| 26 | 1759309_s_at | 4.7 | *dps* | DNA starvation/stationary phase protection protein Dps |
| 27 | 1767843_s_at | 8.0 | *ECs0302* | hypothetical protein |
| 28 | 1760290_s_at | 2.4 | *sucB* | dihydrolipoamide succinyltransferase |
| 29 | 1762757_s_at | 2.0 | *pal* | peptidoglycan-associated outer membrane lipoprotein |
| 30 | 1769013_s_at | 2.2 | *serS* | seryl-tRNA synthetase |
| 31 | 1760008_s_at | 3.8 | *rmf* | ribosome modulation factor |
| 32 | 1765595_s_at | 2.5 | *ompA* | outer membrane protein A |
| 33 | 1768861_s_at | 7.4 | *ECs1203* | antitermination protein Q |
| 34 | 1766843_s_at | 2.2 | *icdA* | isocitrate dehydrogenase |
| 35 | 1765610_s_at | 2.6 | *ycgB* | SpoVR family protein |
| 36 | 1766739_s_at | 2.4 | *ychH* | predicted inner membrane protein |
| **37** | 1764969_s_at | 8.8 | *oppA* | oligopeptide transporter subunit |
| 38 | 1765773_s_at | 2.6 | *oppC* | oligopeptide transport system permease protein oppC |
| 39 | 1765513_s_at | 2.3 | *oppF* | oligopeptide transporter subunit |
| 40 | 1759179_s_at | 2.2 | *osmB* | lipoprotein |
| 41 | 1769101_s_at | 3.3 | *pspA* | phage shock protein PspA |
| 42 | 1762530_s_at | 2.4 | *pspC* | DNA-binding transcriptional activator PspC |
| 43 | 1764975_s_at | 2.1 | *ycjU* | putative beta-phosphoglucomutase |
| 44 | 1761449_s_at | 2.1 | *slyA* | transcriptional regulator SlyA |
| 45 | 1759780_s_at | 3.5 | *lpp* | major outer membrane lipoprotein precursor |
| 46 | 1764443_s_at | 2.2 | *gatB* | galactitol-specific PTS system component IIB |
| 47 | 1762677_s_at | 4.2 | *gatA* | galactitol-specific PTS system component IIA |
| 48 | 1762947_s_at | 5.9 | *gatZ* | putative tagatose 6-phosphate kinase 1 |
| 49 | 1765968_s_at | 2.0 | *rplY* | 50S ribosomal protein L25 |
| 50 | 1764240_s_at | 2.0 | *talA* | transaldolase A |
| 51 | 1764175_s_at | 2.9 | *iscR* | DNA-binding transcriptional regulator IscR |
| 52 | 1760671_s_at | 3.0 | *rpoE* | RNA polymerase sigma factor RpoE |
| 53 | 1765284_s_at | 4.6 | *raiA* | translation inhibitor protein RaiA, cold shock protein associated with 30S ribosomal subunit |
| 54 | 1763652_s_at | 4.3 | *fbaA* | fructose-bisphosphate aldolase |
| 55 | 1760834_s_at | 2.7 | *pitB* | putative low-affinity phosphate transport protein |
| 56 | 1759368_s_at | 2.7 | *smf* | DNA protecting protein DprA |
| 57 | 1767563_s_at | 3.7 | *rpoA* | DNA-directed RNA polymerase subunit alpha |
| 58 | 1762768_s_at | 6.3 | *rpsK* | 30S ribosomal protein S11 |
| 59 | 1761999_s_at | 3.6 | *fusA* | elongation factor G |
| 60 | 1762933_s_at | 2.6 | *rpsG* | 30S ribosomal protein S7 |
| 61 | 1763838_s_at | 2.3 | *rpoH* | RNA polymerase factor sigma-32 |
| 62 | 1765321_s_at | 3.3 | *hdeA* | acid-resistance protein |
| 63 | 1762501_s_at | 2.0 | *fdoH* | formate dehydrogenase-O iron-sulfur subunit |
| 64 | 1765516_s_at | 2.1 | *hflX* | putative GTPase HflX |
| 65 | 1766425_s_at | 2.7 | *priB* | primosomal replication protein N |
| 66 | 1767263_s_at | 2.7 | *eno* | phosphopyruvate hydratase |
| 67 | 1769115_s_at | 2.7 | *fabG* | 3-ketoacyl-(acyl-carrier-protein) reductase |
| 68 | 1764958_s_at | 2.4 | *gatC* | PTS system, galactitol-specific IIC component |
| 69 | 1767672_s_at | 4.6 | *gatY* | tagatose-bisphosphate aldolase |
| 70 | 1760076_at | 3.1 | *glpC* | sn-glycerol-3-phosphate dehydrogenase subunit C |
| 71 | 1768518_s_at | 4.0 | *groEL* | chaperonin GroEL |
| 72 | 1764894_s_at | 2.2 | *groES* | co-chaperonin GroES |
| 73 | 1765901_s_at | 2.2 | *hfq* | RNA-binding protein Hfq |
| 74 | 1761492_s_at | 2.5 | *ihfA* | integration host factor subunit alpha |
| 75 | 1768334_s_at | 3.9 | *infC* | translation initiation factor IF-3 |
| 76 | 1765506_s_at | 2.2 | *livM* | leucine/isoleucine/valine transporter permease subunit |
| 77 | 1764468_s_at | 2.5 | *lpxC* | UDP-3-O-[3-hydroxymyristoyl] N-acetylglucosamine deacetylase |
| 78 | 1762453_s_at | 2.5 | *miaA* | tRNA delta(2)-isopentenylpyrophosphate transferase |
| 79 | 1765424_at | 2.1 | *nikE* | nickel transporter ATP-binding protein NikE |
| 80 | 1764712_s_at | 2.9 | *nlpD* | predicted outer membrane lipoprotein |
| 81 | 1768937_at | 6.4 | *ompC* | outer membrane porin protein C |
| 82 | 1767825_s_at | 3.1 | *ompX* | outer membrane protein X |
| 83 | 1761726_s_at | 7.2 | *oppB* | oligopeptide transporter permease |
| 84 | 1765783_s_at | 3.2 | *oppD* | oligopeptide transporter ATP-binding component |
| 85 | 1768544_at | 2.8 | *papD_2* | PapD protein |
| 86 | 1765539_s_at | 2.8 | *pgk* | phosphoglycerate kinase |
| 87 | 1768435_s_at | 3.2 | *pspB* | phage shock protein B |
| 88 | 1767883_s_at | 5.8 | *rimM* | 16S rRNA-processing protein RimM |
| 89 | 1765939_s_at | 3.2 | *rlmE* | 23S rRNA methyltransferase |
| 90 | 1762846_s_at | 2.0 | *rplA* | 50S ribosomal protein L1 |
| 91 | 1760752_s_at | 2.7 | *rplB* | 50S ribosomal subunit protein L2 |
| 92 | 1765697_s_at | 2.9 | *rplC* | 50S ribosomal protein L3 |
| 93 | 1761917_s_at | 3.3 | *rplD* | 50S ribosomal protein L4 |
| 94 | 1767821_s_at | 3.1 | *rplE* | 50S ribosomal protein L5 |
| 95 | 1766400_s_at | 4.6 | *rplF* | 50S ribosomal protein L6 |
| 96 | 1761300_s_at | 2.1 | *rplK* | 50S ribosomal protein L11 |
| 97 | 1760773_s_at | 4.1 | *rplN* | 50S ribosomal subunit protein L14 |
| 98 | 1764794_s_at | 4.6 | *rplO* | 50S ribosomal protein L15 |
| 99 | 1765986_s_at | 2.0 | *rplQ* | 50S ribosomal protein L17 |
| 100 | 1762255_s_at | 2.4 | *rplR* | 50S ribosomal protein L18 |
| 101 | 1760646_s_at | 2.6 | *rplU* | 50S ribosomal subunit protein L21 |
| 102 | 1767321_s_at | 2.7 | *rplX* | 50S ribosomal protein L24 |
| 103 | 1765749_s_at | 2.6 | *rpmD* | 50S ribosomal protein L30 |
| 104 | 1762036_s_at | 3.5 | *rpmE* | 50S ribosomal protein L31 |
| 105 | 1767807_s_at | 3.5 | *rpmF* | 50S ribosomal protein L32 |
| 106 | 1768595_s_at | 2.6 | *rpmJ* | 50S ribosomal protein L36 |
| 107 | 1762512_s_at | 3.9 | *rpsA* | 30S ribosomal protein S1 |
| 108 | 1765214_s_at | 3.7 | *rpsB* | 30S ribosomal protein S2 |
| 109 | 1765207_s_at | 2.3 | *rpsD* | 30S ribosomal protein S4 |
| 110 | 1765151_s_at | 2.8 | *rpsE* | 30S ribosomal protein S5 |
| 111 | 1760241_s_at | 5.1 | *rpsF* | 30S ribosomal subunit protein S6 |
| 112 | 1768794_s_at | 2.2 | *rpsH* | 30S ribosomal protein S8 |
| 113 | 1767291_s_at | 3.9 | *rpsJ* | 30S ribosomal protein S10 |
| 114 | 1760745_s_at | 4.9 | *rpsL* | 30S ribosomal subunit protein S12 |
| 115 | 1768867_s_at | 2.9 | *rpsM* | 30S ribosomal protein S13 |
| 116 | 1763008_s_at | 4.4 | *rpsN* | 30S ribosomal protein S14 |
| 117 | 1763744_s_at | 2.2 | *rpsO* | 30S ribosomal protein S15 |
| 118 | 1765175_s_at | 4.3 | *rpsP* | 30S ribosomal protein S16 |
| 119 | 1761856_s_at | 2.7 | *rpsS* | 30S ribosomal protein S19 |
| 120 | 1767590_s_at | 5.3 | *rpsV* | 30S ribosomal subunit S22 |
| 121 | 1763891_s_at | 25.0 | *rrfA, rrfB, rrfC, rrfD, rrfE, rrfF, rrfG, rrfH* | 5S ribosomal RNA of rrnA, rrnB, rrnC, rrnD, rrnE, rrnG, rrnH operon |
| 122 | 1760713_s_at | 32.3 | *rrlA, rrlB, rrlC, rrlC, rrlD, rrlE, rrlG, rrlH* | 23S ribosomal RNA of rrnA rrnB, rrnC, rrnD, rrnE, rrnG, rrnH operon |
| 123 | 1766157_s_at | 26.1 | *rrsA, rrsB, rrsC, rrsD, rrsE, rrsG* | 16S ribosomal RNA of rrnA, rrnB, rrnC, rrnD, rrnE, rrnG, operon |
| 124 | 1763456_s_at | 80.9 | *rrsA, rrsB, rrsC, rrsD, rrsE, rrsG rrsH* | 16S ribosomal RNA of rrnH operon |
| 125 | 1767404_s_at | 3.6 | *secY* | preprotein translocase subunit SecY |
| 126 | 1761419_s_at | 9.1 | *speB* | agmatinase |
| 127 | 1769042_s_at | 2.9 | *sucC* | succinyl-CoA synthetase subunit beta |
| 128 | 1761050_s_at | 2.9 | *tnaA* | tryptophanase |
| 129 | 1764493_s_at | 6.9 | *trmD* | tRNA (guanine-N(1)-)-methyltransferase |
| 130 | 1761735_s_at | 2.1 | *wrbA* | TrpR binding protein WrbA |
| 131 | 1763312_at | 55.7 | *csrB* | ncRNA |
| 132 | 1768835_at | 17.9 | *csrC* | ncRNA |
| 133 | 1766397_s_at | 9.9 | *csrC* | ncRNA |
| 134 | 1763985_s_at | 5.0 | *ffs* | ncRNA |
| 135 | 1767680_at | 2.6 | *gcvB* | ncRNA |
| 136 | 1768079_at | 4.6 | *glmY* | ncRNA |
| 137 | 1764678_at | 2.6 | *glmZ* | ncRNA |
| 138 | 1762699_at | 4.2 | *micF* | ncRNA |
| 139 | 1760716_s_at | 11.3 | *rnpB* | ncRNA |
| 140 | 1762278_at | 4.7 | *rybA* | ncRNA |
| 141 | 1760679_at | 9.9 | *ryjA* | ncRNA |
| 142 | 1762568_s_at | 22.4 | *ssrA* | tmRNA |
| 143 | 1763089_s_at | 10.8 | *ssrS* | ncRNA |
| 144 | 1759494_s_at | 3.6 | *bhsA* | hypothetical protein |
| 145 | 1766612_at | 2.1 | *c0339* | hypothetical protein |
| 146 | 1761508_s_at | 2.0 | *c0650* | hypothetical protein |
| 147 | 1761754_s_at | 2.1 | *c1141* | hypothetical protein |
| 148 | 1761825_at | 3.2 | *c1191* | hypothetical protein |
| 149 | 1761672_at | 3.1 | *c2430* | hypothetical protein |
| 150 | 1765596_at | 3.7 | *c2814* | hypothetical protein |
| 151 | 1762989_s_at | 3.0 | *c3151* | hypothetical protein |
| 152 | 1763669_s_at | 2.0 | *c3486* | hypothetical protein |
| 153 | 1768188_at | 3.5 | *c3657* | hypothetical protein |
| 154 | 1768540_at | 5.0 | *c4419* | hypothetical protein |
| 155 | 1759254_at | 7.1 | *c4973* | hypothetical protein |
| 156 | 1768600_s_at | 4.4 | *c5154* | hypothetical protein |
| 157 | 1761112_at | 2.0 | *c5295* | hypothetical protein |
| 158 | 1764543_at | 2.3 | *chuY* | hypothetical protein |
| 159 | 1765036_s_at | 2.9 | *yccV* | hypothetical protein |
| 160 | 1759715_s_at | 3.4 | *yccA* | hypothetical protein |
| 161 | 1763486_s_at | 2.5 | *yceD* | hypothetical protein |
| 162 | 1762658_s_at | 2.0 | *yeaG* | hypothetical protein |
| 163 | 1768481_s_at | 2.2 | *yebV* | hypothetical protein |
| 164 | 1762003_s_at | 2.4 | *yrbL* | hypothetical protein |
| 165 | 1764346_at | 3.1 | *ECs4566* | hypothetical protein |
| 166 | 1763144_at | 3.3 | *yedK* | hypothetical protein |
| 167 | 1765109_at | 2.8 | *yggR* | hypothetical protein |
| 168 | 1767797_at | 2.8 | *yhjX* | hypothetical protein |
| 169 | 1768987_at | 2.8 | *yjeF* | hypothetical protein |
| 170 | 1768179_at | 2.5 | *Z5095* | hypothetical protein |
| 171 | 1759083_at | 2.2 | *-* | unknown |
| 172 | 1760017_at | 3.0 | *-* | unknown |
| 173 | 1766587_s_at | 2.4 | *-* | unknown |
| 174 | 1759110_s_at | 3.6 | *-* | unknown |
| 175 | 1764821_s_at | 2.6 | *-* | unknown |
| 176 | 1765325_s_at | 3.8 | *-* | unknown |
| 177 | 1762912_s_at | 2.2 | *-* | unknown |
| 178 | 1762413_s_at | 3.4 | *-* | unknown |
| 179 | 1764615_s_at | 2.5 | *-* | unknown |

Genes that showed fold change greater than 2.0 (P < 0.05)

**Table S3.** UP regulation of genes in *Escherichia coli* long-term stationary phase cells in LB medium supplemented with glycerol.

| **Sl. No** | **Probe set ID** | **Fold change** | **Gene** | **Gene function** |
| --- | --- | --- | --- | --- |
| 1 | 1763673_s_at | 2.2 | *arsC* | arsenate reductase |
| 2 | 1760246_s_at | 2.5 | *bcsB* | regulator of cellulose synthase, cyclic di-GMP binding |
| 3 | 1766986_s_at | 2.7 | *bioF* | 8-amino-7-oxononanoate synthase |
| 4 | 1764886_s_at | 2.2 | *rzoD, rzoR* | putative Rz endopeptidase from lambdoid prophage DLP12 |
| 5 | 1759677_at | 2.5 | *cdh* | CDP-diacylglycerol pyrophosphatase |
| 6 | 1766403_s_at | 2.1 | *coaA* | pantothenate kinase |
| 7 | 1759826_s_at | 3.6 | *cysH* | 3'-phosphoadenosine 5'-phosphosulfate reductase |
| 8 | 1764181_x_at | 2.0 | *dicF1* | misc_RNA |
| 9 | 1766930_s_at | 3.4 | *ydcC* | H repeat-containing protein |
| 10 | 1767281_s_at | 2.8 | *ydcC* | H repeat-containing protein |
| 11 | 1763291_x_at | 4.0 | *ybfD* | H repeat-containing protein |
| 12 | 1766811_s_at | 2.1 | *sfmC* | putative chaperone |
| 13 | 1766261_x_at | 6.0 | *insC* | putative transposase |
| 14 | 1761216_s_at | 2.1 | *nohB* | terminase small subunit |
| 15 | 1759386_x_at | 2.0 | *hisL* | his operon leader peptide |
| 16 | 1763569_s_at | 2.0 | *rimN* | putative ribosome maturation factor |
| 17 | 1761036_s_at | 2.0 | *yheT* | putative hydrolase |
| 18 | 1766810_s_at | 2.7 | *gntT* | gluconate permease |
| 19 | 1762697_s_at | 2.6 | *xylR* | putative regulator of xyl operon |
| 20 | 1762271_s_at | 2.0 | *uhpC* | regulatory protein UhpC |
| 21 | 1765969_s_at | 2.2 | *yidK* | putative symporter YidK |
| 22 | 1759821_s_at | 3.3 | *ilvG* | acetolactate synthase 2 catalytic subunit |
| 23 | 1760524_s_at | 2.1 | *yifK* | putative transport protein YifK |
| 24 | 1762372_s_at | 2.0 | *rarD* | predicted chloramphenical resistance permease |
| 25 | 1767223_s_at | 2.1 | *hydG* | transcriptional regulatory protein ZraR |
| 26 | 1768453_s_at | 3.0 | *phnE* | membrane channel protein component of Pn transporter |
| 27 | 1759774_at | 2.2 | *entF* | enterobactin synthase multienzyme complex component, ATP-dependent |
| 28 | 1767318_s_at | 2.5 | *exbB* | biopolymer transport protein ExbB |
| 29 | 1763517_s_at | 3.3 | *gor* | glutathione reductase |
| 30 | 1759161_s_at | 2.5 | *hycB* | hydrogenase 3, Fe-S subunit |
| 31 | 1762495_s_at | 9.1 | *insB* | IS1 transposase InsAB' |
| 32 | 1761145_s_at | 6.6 | *insC* | KpLE2 phage-like element; IS2 insertion element repressor InsA |
| 33 | 1759540_s_at | 8.7 | *insC* | transposase insC |
| 34 | 1766923_s_at | 5.8 | *insE* | IS3 element protein InsE |
| 35 | 1759674_s_at | 8.8 | *insD* | insertion element IS2 transposase InsD |
| 36 | 1766257_s_at | 7.4 | *insF* | IS3 element protein InsF |
| **37** | 1764530_s_at | 10.7 | *insF* | IS3 element protein InsF |
| 38 | 1765954_s_at | 10.1 | *insH* | IS5 transposase and trans-activator |
| 39 | 1768300_s_at | 7.0 | *insL* | IS186/IS421 transposase |
| 40 | 1761146_s_at | 4.0 | *livG* | leucine/isoleucine/valine transporter subunit |
| 41 | 1760742_s_at | 2.6 | *lldD* | L-lactate dehydrogenase |
| 42 | 1767514_s_at | 2.5 | *murD* | UDP-N-acetylmuramoyl-L-alanyl-D-glutamate synthetase |
| 43 | 1769023_s_at | 2.0 | *nikA* | nickel-binding periplasmic protein precursor |
| 44 | 1759669_s_at | 2.1 | *nikD* | nickel transporter subunit |
| 45 | 1767357_s_at | 2.1 | *nikE* | nickel transporter ATP-binding protein NikE |
| 46 | 1763447_s_at | 2.1 | *nuoM* | NADH dehydrogenase subunit M |
| 47 | 1764101_s_at | 2.2 | *purH* | bifunctional phosphoribosylaminoimidazolecarboxamide formyltransferase/IMP cyclohydrolase |
| 48 | 1759907_s_at | 2.6 | *pyrD* | dihydro-orotate oxidase, FMN-linked /// dihydroorotate dehydrogenase 2 |
| 49 | 1760855_x_at | 2.2 | *rdlA* | ncRNA |
| 50 | 1769236_s_at | 2.7 | *rhaD* | rhamnulose-1-phosphate aldolase |
| 51 | 1760721_s_at | 3.0 | *srlA* | glucitol/sorbitol-specific enzyme IIC component of PTS |
| 52 | 1766546_at | 2.3 | *wecD* | TDP-fucosamine acetyltransferase |
| 53 | 1760416_at | 2.3 | *yaiT* | pseudo |
| 54 | 1768185_at | 2.8 | *ybfD* | conserved protein |
| 55 | 1763262_at | 2.1 | *ydaU* | Rac prophage; conserved protein |
| 56 | 1768752_at | 2.3 | *yehH* | pseudo |
| 57 | 1767015_at | 3.0 | *yfjV* | pseudo |
| 58 | 1767447_s_at | 8.2 | *yhcE* | pseudo |
| 59 | 1766645_s_at | 3.0 | *yicI* | alpha-xylosidase YicI |
| 60 | 1763462_at | 2.8 | *yihP* | predicted transporter |
| 61 | 1760575_s_at | 2.5 | *yjaA* | conserved protein |
| 62 | 1760801_at | 4.2 | *yjbL* | predicted protein |
| 63 | 1765921_at | 3.3 | *yjfF* | predicted sugar transporter subunit: membrane component of ABC superfamily |
| 64 | 1767771_s_at | 2.3 | *arpB* | hypothetical protein |
| 65 | 1760746_s_at | 2.2 | *c0500* | hypothetical protein |
| 66 | 1761680_s_at | 3.2 | *c0723* | hypothetical protein |
| 67 | 1762972_s_at | 2.7 | *c1304* | hypothetical protein |
| 68 | 1762588_s_at | 2.2 | *c1855* | hypothetical protein |
| 69 | 1761463_s_at | 2.1 | *c2193* | hypothetical protein |
| 70 | 1759795_s_at | 2.7 | *c2375* | hypothetical protein |
| 71 | 1768726_x_at | 2.4 | *c2574* | hypothetical protein |
| 72 | 1759841_s_at | 3.4 | *c2676* | hypothetical protein |
| 73 | 1762094_s_at | 2.7 | *c2918* | hypothetical protein |
| 74 | 1768337_s_at | 6.5 | *c3113* | hypothetical protein |
| 75 | 1761529_s_at | 2.4 | *c3233* | hypothetical protein |
| 76 | 1764154_s_at | 3.3 | *c3466* | hypothetical protein |
| 77 | 1760349_s_at | 6.8 | *yhaB* | hypothetical protein |
| 78 | 1763785_s_at | 2.0 | *c3915* | hypothetical protein |
| 79 | 1764390_s_at | 2.2 | *c4090* | hypothetical protein |
| 80 | 1761612_s_at | 4.2 | *c4174* | hypothetical protein |
| 81 | 1765996_s_at | 2.2 | *c4174* | hypothetical protein |
| 82 | 1760705_at | 2.1 | *c4230* | hypothetical protein |
| 83 | 1767314_s_at | 2.3 | *c4243* | hypothetical protein |
| 84 | 1763059_s_at | 2.2 | *ldrD* | hypothetical protein |
| 85 | 1769122_at | 2.1 | *c4434* | hypothetical protein |
| 86 | 1764646_at | 2.4 | *c4743* | hypothetical protein |
| 87 | 1763971_at | 2.0 | *c4934* | hypothetical protein |
| 88 | 1765967_s_at | 2.4 | *c4938* | hypothetical protein |
| 89 | 1759254_at | 2.2 | *c4973* | hypothetical protein |
| 90 | 1759700_s_at | 2.6 | *ydcD* | hypothetical protein |
| 91 | 1768475_s_at | 3.2 | *rhsA* | hypothetical protein |
| 92 | 1763807_s_at | 2.0 | *ycfT* | hypothetical protein |
| 93 | 1763113_s_at | 2.0 | *yrdB* | hypothetical protein |
| 94 | 1761800_s_at | 2.1 | *tsgA* | hypothetical protein |
| 95 | 1760610_s_at | 2.1 | *yhgA* | hypothetical protein |
| 96 | 1768285_s_at | 2.9 | *yibI* | hypothetical protein |
| 97 | 1768566_at | 2.1 | *ECs4593* | hypothetical protein |
| 98 | 1759376_s_at | 2.2 | *yihD* | hypothetical protein |
| 99 | 1767780_s_at | 2.1 | *ycbC* | hypothetical protein |
| 100 | 1763219_s_at | 2.1 | *yidB* | hypothetical protein |
| 101 | 1766017_s_at | 2.5 | *ydiF* | hypothetical protein |
| 102 | 1768317_s_at | 2.3 | *yjbG* | hypothetical protein |
| 103 | 1765663_s_at | 2.2 | *ykiA* | hypothetical protein |
| 104 | 1764570_s_at | 2.1 | *yneL* | hypothetical protein |
| 105 | 1761899_s_at | 2.2 | *Z0115* | hypothetical protein |
| 106 | 1759349_s_at | 3.5 | *Z2263* | Rhs element protein |
| 107 | 1765215_s_at | 2.4 | *Z4613* | hypothetical protein |
| 108 | 1763319_at | 2.6 | *Z5430* | hypothetical protein |
| 109 | 1762957_s_at | 2.5 | *-* | unknown |
| 110 | 1762241_s_at | 2.1 | *-* | unknown |
| 111 | 1766426_s_at | 2.1 | *-* | unknown |
| 112 | 1768369_s_at | 7.0 | *-* | unknown |
| 113 | 1761584_s_at | 2.2 | *-* | unknown |
| 114 | 1759355_s_at | 6.5 | *-* | unknown |
| 115 | 1765998_s_at | 2.4 | *-* | unknown |
| 116 | 1765228_s_at | 7.9 | *-* | unknown |
| 117 | 1761029_s_at | 2.2 | *-* | unknown |
| 118 | 1767061_s_at | 2.0 | *-* | unknown |
| 119 | 1766644_s_at | 2.0 | *-* | unknown |
| 120 | 1763191_s_at | 2.0 | *-* | unknown |
| 121 | 1763315_s_at | 2.8 | *-* | unknown |
| 122 | 1761069_s_at | 2.2 | *-* | unknown |
| 123 | 1762809_s_at | 2.1 | *-* | unknown |
| 124 | 1764872_s_at | 9.4 | *-* | unknown |
| 125 | 1762731_s_at | 2.2 | *-* | unknown |
| 126 | 1766941_s_at | 2.6 | *-* | unknown |
| 127 | 1768683_s_at | 2.4 | *-* | unknown |
| 128 | 1760195_s_at | 3.5 | *-* | unknown |
| 129 | 1762802_s_at | 3.0 | *-* | unknown |
| 130 | 1761761_s_at | 2.2 | *-* | unknown |
| 131 | 1762591_s_at | 3.0 | *-* | unknown |
| 132 | 1769228_s_at | 2.4 | *-* | unknown |
| 133 | 1766610_s_at | 2.1 | *-* | unknown |
| 134 | 1765877_s_at | 3.4 | *-* | unknown |
| 135 | 1763962_s_at | 2.3 | *-* | unknown |
| 136 | 1762804_s_at | 2.5 | *-* | unknown |
| 137 | 1761721_s_at | 2.5 | *-* | unknown |
| 138 | 1768108_s_at | 3.5 | *-* | unknown |

Genes that showed fold change greater than 2.0 (P < 0.05)

**Table S4.** Down regulation of genes in *Escherichia coli* long-term stationary phase cells in LB medium supplemented with glycerol

| **Sl. No** | **Probe set ID** | **Fold change** | **Gene** | **Gene function** |
| --- | --- | --- | --- | --- |
| 1 | 1766568_s_at | 2.9 | *acpP* | acyl carrier protein |
| 2 | 1761034_at | 3.2 | *aefA* | potassium efflux protein KefA |
| 3 | 1766199_at | 3.8 | *agaS* | putative tagatose-6-phosphate ketose/aldose isomerase |
| 4 | 1767961_s_at | 2.4 | *ahpC* | alkyl hydroperoxide reductase subunit C |
| 5 | 1766031_at | 2.2 | *alr* | alanine racemase |
| 6 | 1766431_s_at | 2.2 | *argH* | argininosuccinate lyase |
| 7 | 1762445_at | 7.0 | *argI* | ornithine carbamoyltransferase subunit F |
| 8 | 1767853_at | 2.2 | *aroC* | chorismate synthase |
| 9 | 1760565_at | 3.3 | *c0336* | PTS system, mannitol (Cryptic)-specific IIA component |
| 10 | 1767207_s_at | 2.0 | *pflB* | pyruvate formate lyase I |
| 11 | 1767353_s_at | 2.1 | *c1459* | putative tail component of prophage |
| 12 | 1765363_s_at | 2.1 | *dhaL* | dihydroxyacetone kinase ADP-binding subunit |
| 13 | 1761180_at | 2.0 | *c1691* | putative gumP-like protein |
| 14 | 1764773_at | 2.2 | *c2420* | putative cytoplasmic transmembrane protein |
| 15 | 1759877_at | 3.1 | *c3406* | phosphosugar isomerase |
| 16 | 1762899_at | 2.8 | *c5038* | putative membrane-bound protein |
| 17 | 1767087_s_at | 2.7 | *cbpA* | curved DNA-binding protein CbpA |
| 18 | 1759918_s_at | 2.8 | *clpA* | ATP-dependent Clp protease ATP-binding subunit |
| 19 | 1762581_at | 2.2 | *cycA* | D-alanine/D-serine/glycine permease |
| 20 | 1763881_s_at | 3.2 | *cydA* | cytochrome d terminal oxidase, polypeptide subunit I |
| 21 | 1765588_s_at | 2.0 | *cydB* | cytochrome d terminal oxidase polypeptide subunit II |
| 22 | 1762134_s_at | 3.1 | *cysG* | siroheme synthase |
| 23 | 1764785_at | 2.4 | *cysP* | thiosulfate transporter subunit |
| 24 | 1759413_at | 3.2 | *cysU* | sulfate/thiosulfate transporter subunit |
| 25 | 1764747_s_at | 2.9 | *dhaK* | dihydroxyacetone kinase, N-terminal domain |
| 26 | 1762471_at | 2.8 | *dppC* | dipeptide transporter |
| 27 | 1761678_s_at | 2.3 | *hrpB* | ATP-dependent RNA helicase HrpB |
| 28 | 1767843_s_at | 5.0 | *ECs0302* | Cnr-like protein |
| 29 | 1766093_s_at | 2.1 | *ybaT* | putative amino acid/amine transport protein |
| 30 | 1768363_s_at | 2.0 | *uspG* | universal stress protein UP12 |
| 31 | 1763338_s_at | 2.1 | *ECs0814* | putative outer membrane protein |
| 32 | 1765296_s_at | 3.1 | *ECs0827* | putative portal protein |
| 33 | 1763071_s_at | 2.3 | *ECs0839* | putative minor tail protein |
| 34 | 1763037_s_at | 2.2 | *poxB* | pyruvate dehydrogenase |
| 35 | 1760008_s_at | 3.7 | *rmf* | ribosome modulation factor |
| 36 | 1765595_s_at | 2.4 | *ompA* | outer membrane protein A |
| **37** | 1765453_s_at | 2.2 | *hyaA* | hydrogenase-1 small subunit |
| 38 | 1767131_at | 2.5 | *ECs1200* | DNA-binding protein |
| 39 | 1768861_s_at | 14.3 | *ECs1203* | antitermination protein Q |
| 40 | 1759490_s_at | 2.1 | *ECs1739* | global DNA-binding transcriptional dual regulator H-NS |
| 41 | 1764969_s_at | 3.7 | *oppA* | oligopeptide transport periplasmic binding protein |
| 42 | 1763696_s_at | 2.5 | *ompW* | outer membrane protein W |
| 43 | 1764975_s_at | 3.7 | *ycjU* | putative beta-phosphoglucomutase |
| 44 | 1762683_s_at | 2.6 | *uspE* | universal stress protein UspE |
| 45 | 1768165_s_at | 2.7 | *gadC* | acid sensitivity protein |
| 46 | 1760545_s_at | 2.6 | *gadB* | glutamate decarboxylase isozyme |
| 47 | 1759780_s_at | 4.2 | *lpp* | murein lipoprotein |
| 48 | 1765823_s_at | 2.2 | *yeaQ* | conserved inner membrane protein |
| 49 | 1767443_s_at | 2.0 | *ECs2639* | putative tail protein |
| 50 | 1764051_s_at | 2.2 | *gatC* | PTS system galactitol-specific enzyme IIC |
| 51 | 1764443_s_at | 2.5 | *gatB* | galactitol-specific PTS system component IIB |
| 52 | 1764719_s_at | 2.3 | *yehY* | putative transport system permease protein |
| 53 | 1764832_s_at | 2.2 | *ECs3029* | putative isomerase-decarboxylase |
| 54 | 1764240_s_at | 2.6 | *talA* | transaldolase A |
| 55 | 1761396_at | 2.6 | *ECs3383* | putative anaerobic dimethyl sulfoxide reductase chain B |
| 56 | 1764370_s_at | 2.6 | *yfiD* | autonomous glycyl radical cofactor GrcA |
| 57 | 1765284_s_at | 4.0 | *raiA* | translation inhibitor protein RaiA |
| 58 | 1761895_s_at | 2.3 | *yhbU* | putative collagenase |
| 59 | 1761999_s_at | 2.1 | *fusA* | elongation factor G |
| 60 | 1765321_s_at | 4.8 | *hdeA* | acid-resistance protein |
| 61 | 1768498_s_at | 2.0 | *gadA* | glutamate decarboxylase isozyme |
| 62 | 1764774_s_at | 2.4 | *phnI* | phosphonate metabolism |
| 63 | 1767263_s_at | 2.7 | *eno* | phosphopyruvate hydratase |
| 64 | 1769115_s_at | 2.0 | *fabG* | 3-ketoacyl-(acyl-carrier-protein) reductase |
| 65 | 1768636_at | 2.8 | *fadA* | 3-ketoacyl-CoA thiolase |
| 66 | 1767885_at | 2.7 | *fliC* | flagellar filament structural protein (flagellin) |
| 67 | 1769188_s_at | 2.9 | *fliR* | flagellar biosynthesis protein FliR |
| 68 | 1760040_at | 2.3 | *flu* | antigen 43 (Ag43) phase-variable biofilm formation autotransporter |
| 69 | 1767672_s_at | 2.0 | *gatY* | tagatose-bisphosphate aldolase |
| 70 | 1763782_at | 2.9 | *gatZ* | putative tagatose 6-phosphate kinase gatZ |
| 71 | 1763372_s_at | 2.3 | *gldA* | glycerol dehydrogenase |
| 72 | 1760076_at | 3.7 | *glpC* | sn-glycerol-3-phosphate dehydrogenase subunit C |
| 73 | 1759829_s_at | 3.0 | *hdeB* | acid-resistance protein |
| 74 | 1761492_s_at | 2.7 | *ihfA* | integration host factor subunit alpha |
| 75 | 1765354_s_at | 2.0 | *ihfB* | integration host factor subunit beta |
| 76 | 1768334_s_at | 2.5 | *infC* | translation initiation factor IF-3 |
| 77 | 1765506_s_at | 3.3 | *livM* | leucine/isoleucine/valine transporter permease subunit |
| 78 | 1762067_at | 3.2 | *mgtA* | magnesium-transporting ATPase MgtA |
| 79 | 1763823_at | 2.6 | *narV* | respiratory nitrate reductase 2 gamma chain |
| 80 | 1765820_s_at | 2.0 | *nepI* | ribonucleoside transporter |
| 81 | 1765424_at | 3.2 | *nikE* | nickel transporter ATP-binding protein NikE |
| 82 | 1768937_at | 6.0 | *ompC* | outer membrane porin protein C |
| 83 | 1768544_at | 5.0 | *papD_2* | PapD protein |
| 84 | 1767892_at | 2.2 | *pepN* | aminopeptidase N |
| 85 | 1759253_at | 2.2 | *perM* | putative permease PerM |
| 86 | 1768993_at | 2.7 | *phnI* | PhnI protein |
| 87 | 1763620_at | 2.0 | *proB* | gamma-glutamyl kinase |
| 88 | 1767883_s_at | 2.2 | *rimM* | 16S rRNA-processing protein RimM |
| 89 | 1767807_s_at | 3.3 | *rpmF* | 50S ribosomal protein L32 |
| 90 | 1765214_s_at | 2.3 | *rpsB* | 30S ribosomal protein S2 |
| 91 | 1760241_s_at | 2.1 | *rpsF* | 30S ribosomal subunit protein S6 |
| 92 | 1760745_s_at | 2.0 | *rpsL* | 30S ribosomal subunit protein S12 |
| 93 | 1763008_s_at | 2.2 | *rpsN* | 30S ribosomal protein S14 |
| 94 | 1767590_s_at | 4.1 | *rpsV* | 30S ribosomal subunit S22 |
| 95 | 1763891_s_at | 36.0 | *rrfA , rrfB, rrfC, rrfD, rrfE, rrfF, rrfG, rrfH.* | 5S ribosomal RNA of rrnA, rrnB, rrnC, rrnD, rrnE, rrnG, rrnH operon |
| 96 | 1760713_s_at | 34.0 | *rrlA, rrlB, rrlC, rrlD rrlE, rrlG, rrlH* | 23S ribosomal RNA of rrnA, rrnB, rrnC, rrnD, rrnE, rrnG, rrnH operon |
| 97 | 1766157_s_at | 21.0 | *rrsA, rrsB, rrsC, rrsD, rrsE, rrsG* | 16S ribosomal RNA of rrnA, rrnB, rrnC, rrnD, rrnE, rrnG operon |
| 98 | 1763456_s_at | 82.5 | *rrsA, rrsB,rrsC,rrsD, rrsE, rrsG, rrsH* | 16S ribosomal RNA of rrnH operon |
| 99 | 1761419_s_at | 4.9 | *speB* | agmatinase |
| 100 | 1759634_s_at | 2.1 | *sufA* | Fe-S cluster assembly protein |
| 101 | 1766101_s_at | 3.7 | *wrbA* | TrpR binding protein WrbA |
| 102 | 1761735_s_at | 3.2 | *wrbA* | TrpR binding protein WrbA |
| 103 | 1765154_at | 2.0 | *yadN* | fimbrial-like protein yadN precursor |
| 104 | 1768865_at | 2.2 | *ycbO* | alkanesulfonate transporter substrate-binding subunit |
| 105 | 1763904_at | 2.5 | *yhdX* | amino-acid ABC transporter permease protein YhdX |
| 106 | 1763312_at | 28.2 | *csrB* | ncRNA |
| 107 | 1768835_at | 15.0 | *csrC* | ncRNA |
| 108 | 1766397_s_at | 8.0 | *csrC* | ncRNA |
| 109 | 1763985_s_at | 4.7 | *ffs* | ncRNA |
| 110 | 1767680_at | 2.3 | *gcvB* | ncRNA |
| 111 | 1762699_at | 2.3 | *micF* | ncRNA |
| 112 | 1760716_s_at | 11.7 | *rnpB* | ncRNA |
| 113 | 1762278_at | 2.2 | *rybA* | ncRNA |
| 114 | 1760679_at | 3.9 | *ryjA* | ncRNA |
| 115 | 1762568_s_at | 25.6 | *ssrA* | tmRNA |
| 116 | 1763089_s_at | 5.6 | *ssrS* | ncRNA |
| 117 | 1763144_at | 3.8 | *yedK* | hypothetical protein |
| 118 | 1765109_at | 4.8 | *yggR* | hypothetical protein |
| 119 | 1768213_s_at | 2.3 | *c0297* | hypothetical protein |
| 120 | 1763141_s_at | 2.0 | *c0320* | hypothetical protein |
| 121 | 1766612_at | 3.2 | *c0339* | hypothetical protein |
| 122 | 1759401_at | 2.2 | *c0428* | hypothetical protein |
| 123 | 1761825_at | 2.9 | *c1191* | hypothetical protein |
| 124 | 1768698_s_at | 2.3 | *c1193* | hypothetical protein |
| 125 | 1760677_at | 2.1 | *c1202* | hypothetical protein |
| 126 | 1768327_at | 2.3 | *c1266* | hypothetical protein |
| 127 | 1764777_s_at | 2.2 | *msyB* | hypothetical protein |
| 128 | 1759684_at | 2.2 | *c1414* | hypothetical protein |
| 129 | 1760968_s_at | 2.6 | *c1415* | hypothetical protein |
| 130 | 1760044_at | 2.7 | *c1452* | hypothetical protein |
| 131 | 1762040_at | 2.0 | *c1472* | hypothetical protein |
| 132 | 1769268_at | 2.5 | *c1495* | hypothetical protein |
| 133 | 1762144_s_at | 2.3 | *c1506* | hypothetical protein |
| 134 | 1761515_at | 4.9 | *c1527* | hypothetical protein |
| 135 | 1759649_at | 2.5 | *c1882* | hypothetical protein |
| 136 | 1761651_at | 2.2 | *c1910* | hypothetical protein |
| 137 | 1762267_at | 2.8 | *c2416* | hypothetical protein |
| 138 | 1761672_at | 4.3 | *c2430* | hypothetical protein |
| 139 | 1761350_at | 3.2 | *c2501* | hypothetical protein |
| 140 | 1764307_at | 2.5 | *c2610* | hypothetical protein |
| 141 | 1766789_at | 2.6 | *c2729* | hypothetical protein |
| 142 | 1765596_at | 5.9 | *c2814* | hypothetical protein |
| 143 | 1763800_at | 2.7 | *c3401* | hypothetical protein |
| 144 | 1766566_s_at | 2.0 | *c3507* | hypothetical protein |
| 145 | 1764841_s_at | 2.0 | *c3629* | hypothetical protein |
| 146 | 1768188_at | 4.3 | *c3657* | hypothetical protein |
| 147 | 1760635_at | 2.0 | *c4205* | hypothetical protein |
| 148 | 1768540_at | 2.1 | *c4419* | hypothetical protein |
| 149 | 1768904_s_at | 2.6 | *c4567* | hypothetical protein |
| 150 | 1763254_at | 3.0 | *c4576* | hypothetical protein |
| 151 | 1760018_at | 2.0 | *c5143* | hypothetical protein |
| 152 | 1768600_s_at | 5.8 | *c5154* | hypothetical protein |
| 153 | 1764543_at | 3.2 | *chuY* | hypothetical protein |
| 154 | 1767675_s_at | 2.2 | *ECs0348* | hypothetical protein |
| 155 | 1769106_s_at | 2.4 | *yccJ* | hypothetical protein |
| 156 | 1769156_s_at | 2.3 | *ECs1293* | hypothetical protein |
| 157 | 1767477_x_at | 2.1 | *ECs1367* | hypothetical protein |
| 158 | 1760122_s_at | 2.1 | *ECs1508* | hypothetical protein |
| 159 | 1769035_s_at | 2.1 | *ECs1547* | hypothetical protein |
| 160 | 1760907_s_at | 2.2 | *ECs1963* | hypothetical protein |
| 161 | 1762658_s_at | 2.1 | *yeaG* | hypothetical protein |
| 162 | 1765160_s_at | 2.2 | *ECs2767* | hypothetical protein |
| 163 | 1767757_s_at | 2.7 | *yfeD* | hypothetical protein |
| 164 | 1764293_s_at | 2.0 | *yqjD* | hypothetical protein |
| 165 | 1764346_at | 5.0 | *ECs4566* | hypothetical protein |
| 166 | 1767763_at | 2.1 | *ECs4968* | hypothetical protein |
| 167 | 1760086_s_at | 2.3 | *ECs5415* | hypothetical protein |
| 168 | 1767797_at | 4.0 | *yhjX* | hypothetical protein |
| 169 | 1764383_at | 2.3 | *yidE* | hypothetical protein |
| 170 | 1759273_at | 2.1 | *yjbB* | hypothetical protein |
| 171 | 1768987_at | 7.6 | *yjeF* | hypothetical protein |
| 172 | 1759267_at | 2.2 | *Z0341* | hypothetical protein |
| 173 | 1768549_at | 2.1 | *Z1420* | hypothetical protein |
| 174 | 1768179_at | 3.0 | *Z5095* | hypothetical protein |
| 175 | 1759415_at | 2.2 | *Z6017* | putative transposase |
| 176 | 1765935_s_at | 2.2 | *-* | Unknown |
| 177 | 1760017_at | 3.3 | *-* | Unknown |
| 178 | 1766218_s_at | 2.4 | *-* | Unknown |
| 179 | 1763075_at | 2.2 | *-* | Unknown |
| 180 | 1759110_s_at | 2.4 | *-* | Unknown |
| 181 | 1765325_s_at | 2.5 | *-* | Unknown |
| 182 | 1762413_s_at | 2.9 | *-* | Unknown |
| 183 | 1764615_s_at | 2.2 | *-* | Unknown |

Genes that showed fold change greater than 2.0 (P < 0.05)
